# Effectiveness, immunogenicity, and safety of COVID-19 vaccines for individuals with hematological malignancies: a systematic review

**DOI:** 10.1038/s41408-022-00684-8

**Published:** 2022-05-31

**Authors:** Vanessa Piechotta, Sibylle C. Mellinghoff, Caroline Hirsch, Alice Brinkmann, Claire Iannizzi, Nina Kreuzberger, Anne Adams, Ina Monsef, Jannik Stemler, Oliver A. Cornely, Paul J. Bröckelmann, Nicole Skoetz

**Affiliations:** 1 Evidence-based Oncology, Department I of Internal Medicine, Center for Integrated Oncology Aachen Bonn Cologne Duesseldorf, Faculty of Medicine and University Hospital Cologne, University of Cologne, Cologne, Germany; 2 Department I of Internal Medicine, Center for Integrated Oncology Aachen Bonn Cologne Duesseldorf (CIO ABCD), Faculty of Medicine and University Hospital Cologne, University of Cologne, Cologne, Germany; 3 German Centre for Infection Research (DZIF), partner site Bonn-Cologne, Cologne, NRW Germany; 4 Institute of Medical Statistics and Computational Biology, Faculty of Medicine and University Hospital Cologne, University of Cologne, Cologne, Germany; 5 University of Cologne, Faculty of Medicine and University Hospital Cologne, Clinical Trials Centre Cologne (ZKS Köln), Cologne, Germany; 6 Max-Planck Institute for the Biology of Ageing, Cologne, Germany

**Keywords:** Epidemiology, Clinical trial design

## Abstract

The efficacy of SARS-CoV-2 vaccination in patients with hematological malignancies (HM) appears limited due to disease and treatment-associated immune impairment. We conducted a systematic review of prospective studies published from 10/12/2021 onwards in medical databases to assess clinical efficacy parameters, humoral and cellular immunogenicity and adverse events (AE) following two doses of COVID-19 approved vaccines. In 57 eligible studies reporting 7393 patients, clinical outcomes were rarely reported and rates of SARS-CoV-2 infection (range 0–11.9%), symptomatic disease (0–2.7%), hospital admission (0–2.8%), or death (0–0.5%) were low. Seroconversion rates ranged from 38.1–99.1% across studies with the highest response rate in myeloproliferative diseases and the lowest in patients with chronic lymphocytic leukemia. Patients with B-cell depleting treatment had lower seroconversion rates as compared to other targeted treatments or chemotherapy. The vaccine-induced T-cell response was rarely and heterogeneously reported (26.5–85.9%). Similarly, AEs were rarely reported (0–50.9% ≥1 AE, 0–7.5% ≥1 serious AE). In conclusion, HM patients present impaired humoral and cellular immune response to COVID-19 vaccination with disease and treatment specific response patterns. In light of the ongoing pandemic with the easing of mitigation strategies, new approaches to avert severe infection are urgently needed for this vulnerable patient population that responds poorly to current COVID-19 vaccine regimens.

## Introduction

The ongoing coronavirus disease 2019 (COVID-19) pandemic causes immense mortality and morbidity [1]. Patients with hematological malignancies (HM) have a higher risk of infection after exposure, a worse prognosis after infection, and a higher risk of severe or critical disease and COVID-19-related complications due to disease and/or treatment associated with complex immune dysfunction [2–5].

Vaccines were shown to be a key element to prevent severe diseases resulting in hospitalization and death in most patients [6]. Current evidence proves impaired vaccine-induced immune response in immunocompromised individuals with HM [7, 8]. At the same time, data regarding vaccination to mitigate sequelae by SARS-CoV-2 variants of concern (VOC) is indicative of reduced neutralizing activity and protective efficacy of vaccination, compared to the wild type or less pathogenic variants [9, 10]. Ongoing studies evaluating booster vaccinations and the aim to develop adjusted vaccines addressing VOCs are of utmost importance—especially when considering their impact on vulnerable patient groups, such as immunocompromised individuals [11].

COVID-19 vaccine immunity is mediated by the interplay of humoral and cellular components [12]. In many HM patients a disease-induced dysfunction of the innate and adaptive immune system, as well as a treatment-related immune deficiency, severely impacts the immune response following COVID-19 vaccination. Mechanisms underlying protection against COVID-19 are not yet fully understood. There is already strong evidence that the humoral response is required to prevent infection, while the cellular response seems to be critical for the prevention of severe disease. Yet, the humoral response may not always correlate with T-cell-mediated immunity in immunocompromised patients [13–15]. Data evaluating the vaccine-induced immune response is generally scarce for HM patients since most clinical trials initially excluded such patients. In the past months, several studies have investigated vaccine-induced immunogenicity in patients with cancer and indicated a decreased immune response to COVID-19 vaccines. A better understanding of the immune response to SARS-CoV-2 vaccination in these individuals is critical for optimizing vaccination programs, identifying particularly vulnerable patient groups and areas of unmet need, and thereby reducing severe disease and mortality.

As people with HM are at high risk of severe disease after infection with SARS-CoV-2 [16], and research on vaccine effectiveness in this population is generally scarce, there is an urgent ongoing need to evaluate the effectiveness, immunogenicity, and safety of COVID-19 vaccines for these individuals. There are several studies reporting on the effects of COVID-19 vaccines in hematological and oncological patients [17–21]. However, a comprehensive synthesis of the available evidence and evaluation of patient-relevant outcomes is needed to support disease prevention and the identification of optimized vaccination schedules. Thus, the objective of this systematic review is to assess the effectiveness, immunogenicity, and safety of COVID-19 vaccines for individuals with HM.

## Methods

The protocol for this review was registered with PROSPERO (CRD42021281412) and is provided at Open Science Framework under https://osf.io/2nbev/.

### Literature search

On December 10, 2021, the Web of Science Core collection, the WHO COVID-19 Global literature on Coronavirus disease, and Cochrane COVID-19 Study Register (CCSR)were searched. The search strategies can be found in the supplement. In addition, reference lists of included publications were hand searched.

### Study selection criteria

We included randomized controlled trials and prospective cohort studies, examining adult participants with a diagnosis of a HM who completed vaccination schedules with one or more of the COVID-19 vaccines that have been authorized for use in the European Union or approved, authorized, licensed or granted an emergency use authorization in at least ten countries worldwide; as of September 1, 2021; those were:

- mRNA-based vaccines: BNT162 (Comirnaty®) from Pfizer/BiontechmRNA-1273 (Spikevax®) from Moderna;
- Vector-based vaccines: AZD1222 (Vaxzevria®) from Astrazeneca, JNJ-78436735 (COVID-19 Vaccine Janssen®) from Janssen Pharmaceuticals, rAd26 (Sputnik Light®) from Gamaleya, rAd5 (Sputnik V®, Gam-COVID-Vac®) from Gamaleya;
- Inactivated vaccines: BBIBP-CorV (Covilo®) from Sinopharm, CoronaVac (Sinovac®) from Sinovac Biotech [22].

Complete vaccination (=full primary immunization) schedules were defined as per marketing authorization at the time of study conduct and included two doses for full primary immunization (except COVID-19 Vaccine Janssen®). An additional dose at ≥3 months after completion of full primary immunization was considered as a booster dose. An additional dose <3 months after completion of full primary immunization was considered as optimization of the full primary immunization schedule.

We did not exclude studies based on publication format, as long as sufficient information was available, and the abstract could be retrieved in English. Study selection was performed in duplicate using a web-based online platform (Rayyan; www.rayyan.ai) by VP, CH, AB, CI, and/or NK. Disagreements were resolved by discussion or by consulting a third author [23].

### Data extraction and synthesis of the evidence

Data were extracted by one reviewer and verified by another (VP, CH, CI, AB, NK, SM, and/or PJB). Predefined outcomes were clinical parameters (COVID-19 related mortality, COVID-19-related admission to intensive care unit (ICU), COVID-19 related hospitalization, symptomatic COVID-19, SARS-CoV-2 infection, time to infection, and transmissibility); immunity parameters describing the seroconversion and binding immunoglobulin G titers, seroconversion described by neutralization assays, and T-cell parameters; and adverse events, serious adverse events, and events of special interest (i.e., allergic reactions, thrombotic events, heart muscle inflammation, the progress of underlying malignancy).

We collected outcome data (event frequencies and sample size) for the overall cohort and relevant subgroups and recalculated confidence intervals where necessary. Data on healthy controls of the same studies were extracted, but not directly compared to the HM cohorts, as most of these controls were not well-matched in age and clinical condition. Due to the large clinical, geographical, and temporal heterogeneity of studies, outcome rates were not pooled but depicted in form of forest plots without a combined effect estimate using R (version 4.1.2) as well as presented in a table format, both according to overall identified studies and subgroups (type of disease, type of therapy, age, the biological sex of participants).

### Risk of bias assessment and GRADEing of the evidence

For risk of bias assessments, we used a tool that is currently being developed for overall prognosis studies (RoB-OPS; https://osf.io/dfk2r) and which is based on previous items in prognosis and risk of bias research but tailored to studies without a control arm. Based on the four domains of participants, outcomes, analysis, and reporting bias, an overall judgement of each outcome set was made per study. The complete list of items and the rating options in their current form can be found in the supplement.

For prioritized outcomes, we used the Grading of Recommendations Assessment, Development, and Evaluation (GRADE) approach to assess the certainty in the evidence, starting at a high level of evidence according to guidelines for overall prognosis studies [24, 25].

## Results

### Results of the search

**Fig. 1:**
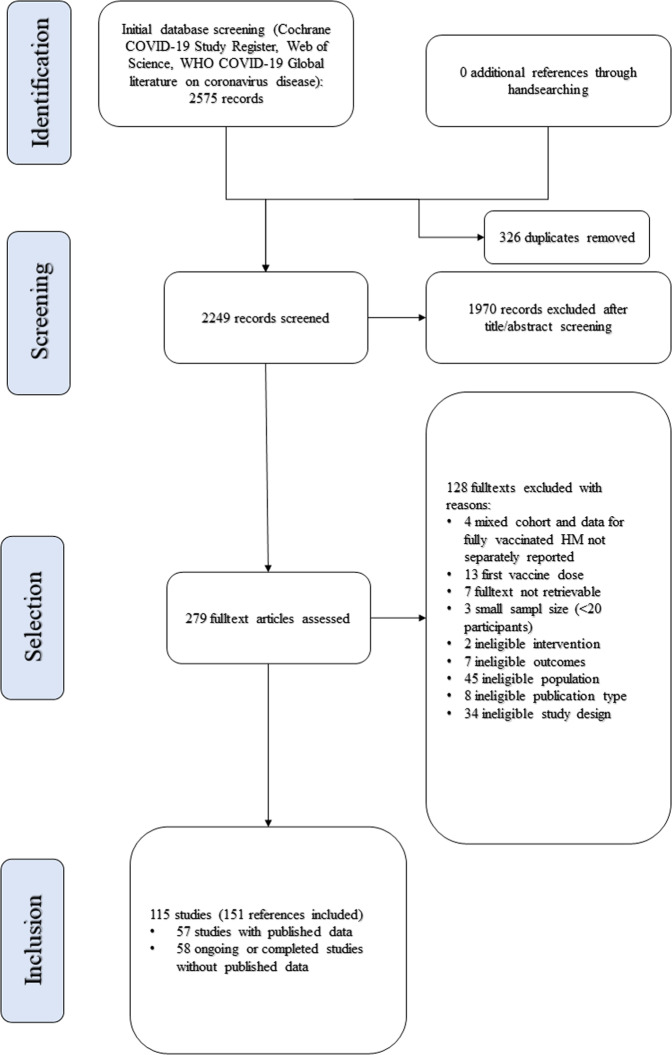
PRISMA flow diagram. Selection process for articles included in systematic review.

The search flow is summarized in Fig. 1. We initially identified 2575 potentially relevant records. After removing 326 duplicates and 1970 records through title and abstract screening, 279 full-text manuscripts were evaluated for eligibility. Further, 128 records were excluded for specific reasons (see Supplementary Table 1). Finally, 156 studies (151 records) that met the inclusion criteria were included. Of those, 58 studies were still ongoing or completed, but without published data (see Supplementary Table 2). After all, 57 studies were included in our outcome synthesis.

### Description of studies

Of the 57 identified studies, all were conducted between December 2020 and October 2021. Most studies were conducted in Europe (29 of 57 studies), followed by 14 studies from the US, and 12 from Israel. We did not identify any information on study location for the two studies. The majority of studies received funding from academic organizations, charities or foundations, or governmental institutions (29 of 57 studies). Eight studies received no dedicated funding, one study was funded by the pharmaceutical industry and the remaining did not disclose funding sources.

Ten studies included only individuals with plasma cell neoplasms (primarily multiple myeloma, MM), four only individuals with chronic lymphocytic leukemia (CLL), and one only individual with Waldenström macroglobulinemia. The remaining studies included participants with different malignancies or did not specify the underlying type of HM. In total, 39 studies had a control group, with 15 of those consisting of healthy volunteers, 12 healthy health care workers (HCW), 3 age-matched or -compatible cohorts, and 2 age- and sex-matched HCW. In two studies the control group consisted of a solid tumour cohort and a non-vaccinated COVID-19-positive MM patient cohort, respectively.

Overall, we identified studies on five different vaccines, which were examined in various combinations: Comirnaty®, Spikevax®, Vaxzevria®, COVID-19 Vaccine Janssen®, and BBIBP-CorV, with Comirnaty® used as a single vaccine in 31/57 studies. Most studies evaluated the effectiveness of a defined full primary immunization schedule (i.e., one dose for COVID-19 Vaccine Janssen®, and two doses for all other vaccines). Two studies evaluating the effect of a third vaccine dose were identified. None of the studies had a prospectively planned interventional comparison. However, five studies enrolled participants receiving different vaccines and provided subgroup outcome data.

All of the included studies reported on at least one immunity parameter of interest. Clinical outcomes evaluating the effectiveness of the vaccines were reported in 22 of 57 studies, and safety outcomes, including data on reactogenicity, were reported in 20 of the studies. Supplementary Table 3 summarizes details regarding the study and participant characteristics.

### Risk of bias

#### Effectiveness outcomes

From the 22 studies reporting clinical outcomes, one study was separately rated for three different clinical outcomes. Among these 24 judgments, the overall risk of bias was rated to be low in three studies, to be moderate in ten studies and to be high in 11 studies. For nine studies no information was available to assess selective reporting bias. The separate judgements for each domain are illustrated in Supplementary Fig. 1, and supporting judgements are available from the authors upon request.

#### Immunogenicity outcomes

From the 57 studies reporting immunogenicity outcomes, three studies were separately rated for two different immunogenicity outcomes. Among these 60 judgements, the overall risk of bias was rated to be low in ten studies, to be moderate in 36 studies and to be high in 14 studies. For nine studies no information was available to assess selective reporting bias. The separate judgements for each domain are illustrated in Supplementary Fig. 2.

#### Safety outcomes

From the 20 studies reporting safety outcomes, one study was separately rated for two different safety outcomes. Among these 21 judgements, the overall risk of bias was rated to be low in none of the studies, to be moderate in nine studies and to be high in 12 studies. For seven studies no information was available to assess selective reporting bias. The separate judgements for each domain are illustrated in supplementary fig. 3.

#### Effectiveness, immunogenicity, and safety of vaccination

A summary of findings with the GRADE-certainty assessments, for outcomes prioritized as most patient-relevant at the protocol stage, is available in Table 1. Further, an overview of all reported outcomes, including the ranges of reported effect rates, and the number of participants and studies is available in Supplementary Table 6.

**Table 1:** Summary of findings.

Population: patients with hematological malignancies Intervention: full primary immunization with SARS-CoV-2 vaccine (i.e., one dose for COVID-19 Vaccine Janssen®, and two doses for all other vaccines)									
Outcome	Certainty assessment						Summary of findings		
	Study design	Risk of bias	Inconsistency	Indirectness	Imprecision	Other considerations	No. of patients (studies)	Effect (Event or response rate)	Certainty
SARS-CoV-2 infection	Observational studies^a^	Serious^b^	No serious	Very serious^c^	No serious	No serious	3277 (19)	0–11.9%	⊕⊖⊖⊖ Very low
Symptomatic COVID-19	Observational studies^a^	Serious^b^	No serious	Very serious^c^	No serious	No serious	1484 (13)	0–2.7%	⊕⊖⊖⊖ Very low
COVID-19-related hospitalization	Observational studies^a^	Serious^b^	No serious	Very serious^c^	Serious^d^	No serious	382 (6)	0–2.8%	⊕⊖⊖⊖ Very low
COVID-19-related mortality	Observational studies^a^	Serious^b^	No serious	Very serious^c^	No serious	No serious	1228 (6)	0–0.5%	⊕⊖⊖⊖ Very low
Antibody response	Observational studies^a^	Serious^b^	Serious^e^	No serious	No serious	No serious	7393 (48)	38.1–99.1%	⊕⊕⊖⊖ Low
T-cell response	Observational studies^a^	Serious^b^	Serious^e^	No serious	No serious	No serious	970 (11)	26.5–79.3%	⊕⊕⊖⊖ Low
Adverse events (any grade)	Observational studies^a^	Serious^b^	Serious^e^	No serious	No serious	No serious	853 (6)	0–50.9%	⊕⊕⊖⊖ Low
Serious adverse events	Observational studies^a^	Serious^b^	No serious	No serious	No serious^d^	No serious	901 (4)	0–7.5%	⊕⊕⊖⊖ Low

SARS-CoV-2 severe acute respiratory syndrome coronavirus type 2, COVID-19 coronavirus disease 2019.

^a^Started with high level of evidence according to guidelines for overall prognosis studies.

^b^Downgraded one level due to serious limitations in most included studies.

^c^Downgraded two levels due to very short observation period, not allowing sufficient time to observe vaccine effectiveness.

^d^Downgraded one level due to small information size.

^e^Downgraded one level due to serious heterogeneity.

##### SARS-CoV-2 infection and severity

**Fig. 2:**
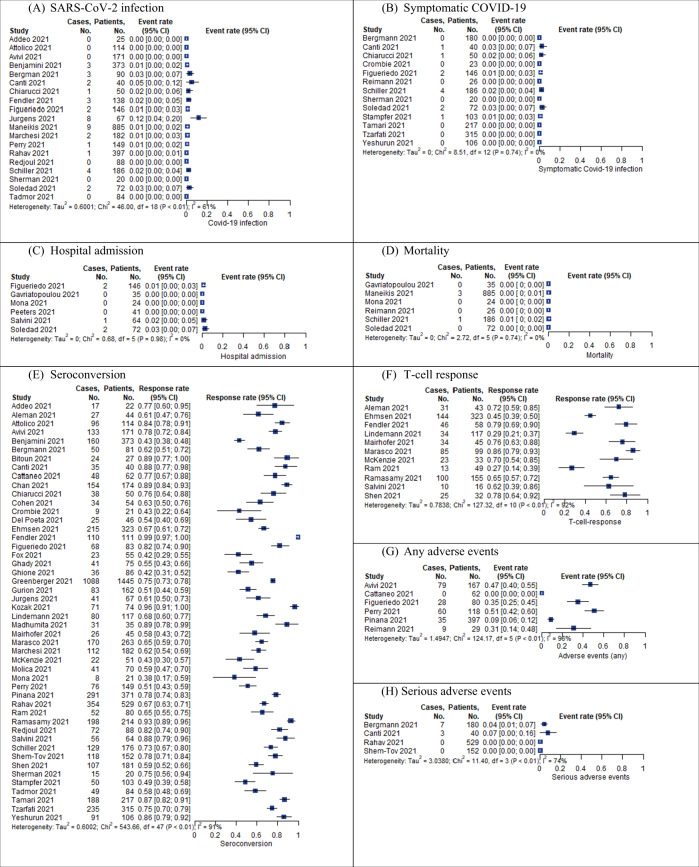
Event and response rate for prioritized outcomes per study. **A** SARS-CoV-2 infection, **B** symptomatic COVID-19, **C** hospital admission, **D** mortality, **E** seroconversion, **F** T-cell response, **G** any adverse events, **H** serious adverse events.

We identified 19 studies (3277 participants with HM) reporting on SARS-CoV-2 infections following full primary immunization. The event rate ranged across studies from 0 to 11.9% (⊕⊖⊖⊖ very-low certainty of the evidence) (see Fig. 2A). The time of follow-up (FU) was not reported in six studies [26–31], and ranged from 11 days to 6 months after the second vaccination in the remaining studies. One study (72 participants with HM) reported the time to occurrence of SARS-CoV-2 infections after full primary immunization. The study reported two cases that occurred after a median 19.5 days (range 16–23) following the second vaccination. It was not reported whether both individuals achieved an immune response after vaccination.

We identified 13 studies (1484 participants with HM) reporting on symptomatic COVID-19. The event rate ranged across studies from 0 to 2.7% (⊕⊖⊖⊖ very-low certainty of the evidence) (see Fig. 2B). FU time was not reported in six studies [26–31] and ranged from 19 days to 6 months after the second vaccination in the remaining studies.

Six studies (382 participants with HM) reporting on COVID-19 related hospitalization (Mona 2021) were identified. The event rate ranged studies from 0 to 2.8% across studies (⊕⊖⊖⊖ very-low certainty of the evidence) (see Fig. 2C). FU time was not reported in three studies [28, 32, 33] one had a follow-up of 2 months, and two studies of 3 months [34, 35] after the second vaccination. One study (72 participants with HM) reported on COVID-19 related admission to ICU. After a follow-up of 3 months after the second vaccination, no cases were observed [35].

In total, we identified six studies (1228 participants with HM) reporting on COVID-19-related mortality in vaccinated individuals. The event rate ranged across studies from 0 to 0.5% (⊕⊖⊖⊖ very-low certainty of the evidence) (see Fig. 2D). We did not identify any studies reporting on transmissibility (e.g., secondary attack rates) or quality of life after SARS-CoV-2 vaccination.

#### Immunogenicity

##### Humoral response

**Fig. 3:**
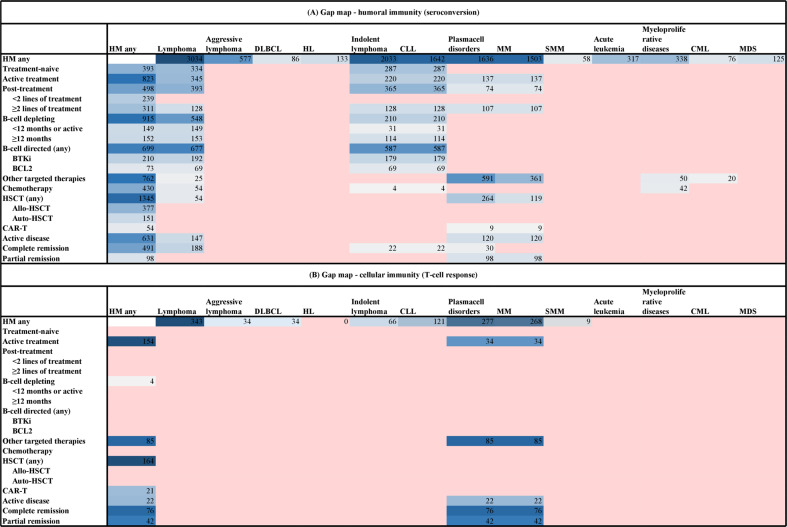
Evidence-gap maps. **A** Humoral immunity (seroconversion), **B** cellular immunity (T-cell response).

Forty-eight studies reported on antibody response after full primary vaccination. Of those, 18 compared their results to a healthy control group and described significant between-group differences in antibody response. Positive humoral immune response was defined in the primary studies according to cutoffs defined for the respective assays used (see Supplementary Table 6). Across studies (including 7393 individuals), response rates ranged from 38.1 to 99.1% after the first measurement at least 14 days after the second vaccine (⊕⊕⊖⊖ low certainty of the evidence) (see Fig. 2E). We stratified the results along underlying disease and treatment received (see Table 2). Detailed results with the number of patients and events are depicted in the corresponding forest plots Supplementary Figs. 4 and 5, respectively. The highest response rate was seen for patients with myeloproliferative diseases and the lowest in patients with CLL. Patients with lymphoma showed very diverse responses irrespective of whether indolent or aggressive lymphoma was reported. Regarding treatment, patients with B-cell depleting or -directed treatment had lower seroconversion rates as compared to those receiving chemotherapy or other targeted treatments. Patients who underwent either allogeneic or autologous stem cell transplantation (SCT) showed higher seroconversion rates than those after CAR T-cell therapy. Available data differed in quantity and detail with regard to different underlying diseases (Fig. 3A). Most data were available for patients with indolent lymphoma, especially MM, and CLL, while data for patients with acute leukaemia and myeloproliferative diseases are sparse. Due to different assays applied in the various studies including heterogeneity in target measurement throughout the studies, the direct comparison of IgG titers was unfeasible and is not reported.

**Table 2:** Humoral immunity (seroconversion).

Population	Response rates	Number of participants (studies)
Healthy controls	96–100%	1836 (18)
HM (any)	38.1–99.1%	7393 (48)
Female sex	43.1–96.0%	725 (11)
Male sex	50.0–84.1%	1004 (11)
Age < 70 years	47.6–87.5%	732 (8)
Age ≥ 70 years	37.0–72.7%	359 (4)
*Type of disease*		
Lymphoma	22.9–100%	3034 (22)
Aggressive lymphoma	41.9–100%	577 (13)
DLBCL	78.9–85.3%	86 (2)
HL	50.0–100%	133 (8)
Indolent lymphoma	42.9–100%	2033 (14)
CLL	42.9–100%	1642 (13)
Plasma cell disorders	0.0–95.8%	1636 (16)
Myeloma	0.0–95.5%	1503 (16)
SMM	90.0–100%	58 (4)
Acute leukemias	45.5–92.0%	317 (7)
AML	42.9–91.2%	116 (4)
ALL	25.0–100%	50 (5)
Myeloproliferative diseases	68.8–97.1%	338 (6)
CML	90.9–100%	76 (3)
MDS	78.3–100%	125 (6)
*Therapy status*		
Treatment naive/ watch-and-wait	61.4–100%	393 (8)
Active treatment	7.3–86.0%	823 (12)
Post-treatment, currently no active treatment	55.6–100%	498 (9)
<2 lines of treatment	78.2%	239 (1)
≥2 lines of treatment	29.5–84.1%	311 (4)
*Type of therapy*		
B-cell depleting (i.e., any anti-CD20, including with chemo)	0.0–88.9%	915 (14)
<12 months or active	0.0–22.2%	149 (6)
≥12 months	34.8–81.8%	152 (4)
B-cell directed (any)	7.7–52.4%	699 (11)
BTKi	7.7–50.0%	210 (8)
BCL2	0.0–25.0%	73 (4)
Other targeted therapies/novel agents	0.0–100%	762 (13)
Chemotherapy	25.0–85.7%	430 (7)
HSCT (any)	68.4–89.0%	1345 (14)
allo-HSCT	50.0–89.3%	377 (5)
auto-HSCT	86.8–94.3%	151 (3)
CART	0.0–77.8%	54 (6)
*Disease status*		
Active disease	65.3–88.1%	631 (5)
Complete remission	27.3–77.9%	491 (5)
Partial remission	80.6%	98 (1)
*Type of disease and therapy (only subgroups with data shown)*		
*Lymphoma*		
Any HSCT	79.6%	54 (1)
B-cell depleting	22.2–88.9%	548 (10)
B-cell depleting ≥12 months or active	34.8–81.8%	153 (4)
B-cell depleting <12 months or active	0.0–22.2%	149 (6)
B-cell directed (any)	7.7–52.4%	677 (9)
BCL2	0.0–24.2%	69 (3)
BTKI	7.7–50.0%	192 (6)
Chemotherapy	25.0–46.0%	54 (2)
Other targeted therapies/novel agents	40%	25 (1)
*Indolent lymphoma CLL*		
B-cell depleting	22.2–88.9%	210 (4)
B-cell depleting ≥12 months or active	34.8–81.8%	114 (2)
B-cell depleting <12 months or active	0.0–22.2%	31 (2)
B-cell directed (any)	16.0–52.4%	587 (7)
BCL2	0.0–24.2%	69 (3)
BTKI	14.3–50.0%	179 (5)
Chemotherapy	25.0%	4 (1)
*Plasma cell disorders*		
Any HSCT	75.9–80.0%	264 (3)
CART	77.8%	9 (1)
Other targeted therapies/novel agents	0.0–90.1%	591 (7)
*MM*		
Any HSCT	76.2–80.0%	119 (2)
CART	77.8%	9 (1)
Other targeted therapies/novel agents	0.0–90.1%	361 (5)
*Myeloproliferative disease*		
Other targeted therapies/novel agents	66.7–100%	50 (2)
Chemotherapy	85.7%	42 (1)
*CML*		
Other targeted therapies/novel agents	100%	20 (1)
*Type of disease and therapy status (only subgroups with data shown)*		
*Lymphoma*		
Treatment naive or watch and wait	61.4–100%	334 (7)
Active treatment	7.3–45.6%	345 (7)
Post-treatment or no active treatment	55.6–79.0%	393 (6)
≥2 lines of therapy	29.5–57.6%	128 (2)
*Indolent lymphoma CLL*		
Treatment naive or watch and wait	61.4–84.6%	287 (4)
Active treatment	17.7–42.9%	220 (5)
Post-treatment or no active treatment	55.6–68.3%	365 (4)
≥2 lines of therapy	29.5–57.6%	128 (2)
*Plasma cell disorders*		
* MM*		
Active treatment	66.4%	137 (1)
Post-treatment or no active treatment	97.1–97.5%	74 (2)
≥2 lines of therapy	84.1%	107 (1)
*Type of disease and disease status (only subgroups with data shown)*		
*Lymphoma*		
Active disease	65.3%	147 (1)
Complete remission	27.3–69.9%	188 (2)
*Indolent lymphoma CLL*		
Complete remission	27.3%	22 (1)
*Plasma cell disorders*		
Active disease	69.4–88.1%	120 (2)
Complete remission	60%	30 (1)
Partial remission	80.6%	98 (1)
* MM*		
Active disease	69.4–88.1%	120 (2)
Partial remission	80.6%	98 (1)

*ALL* acute lymphoblastic leukemia, *allo* allogeneic, *AML* acute myeloid leukemia, *auto* autologous, *BCL2* B-cell lymphoma 2 inhibitor, *BTKI* bruton tyrosine kinase inhibitor, *CART* chimeric antigen receptor (CAR) T-cell therapy, *CLL* chronic lymphocytic leukemia, *CML* chronic myeloid leukemia, *DLBCL* diffuse large B-cell lymphoma, *HL* Hodgkin lymphoma, *HM* hematological malignancy, *HSCT* hematopoietic stem cell transplantation, *MDS* myelodysplastic syndrome, *MM* multiple myeloma, *SMM* smoldering myeloma.

We identified five studies reporting on FU after the second vaccine dose. During the initial measurement, 573 participants with HM were evaluated while at the extended FU measurement 373 participants were evaluated. Response rate at the initial measurement ranged from 58 to 82% and after the extended FU from 45 to 76%, with four studies suggesting a waning IgG antibody response over time (Supplementary Fig. 6). Measurements were performed ~2–3.5 months apart.

Two studies reported IgG response after a third (i.e., “booster”) vaccine dose for a total of 78 participants with HM. The response rate ranged from 31 to 65%. For one study, IgG measurements after the second dose were also available, however for a larger cohort. In comparison, the response rate for the full cohort was 75% after completing full primary immunization and 65% for the patients receiving a booster dose (Supplementary Fig. 7) [36].

Five studies (429 participants with HM) compared IgG antibody responses for different vaccine types. Four studies compared BNT162b2 with mRNA-123 [30, 37–39] and one study BNT162b2 with ChAdox-nCoV-19 [40]. Confidence intervals were widely overlapping for all but one study that compared BNT162b2 with mRNA-123 (27% (95% CI 15–40) vs. 62% (95% CI 49–76)). Further, the one study comparing BNT162b2 with ChAdox-nCoV-19 suggests no difference in response (supplementary fig. 8).

Eighteen studies provided data on healthy controls. As described above, no direct comparisons to the HM cohorts were made, as most of these controls were not well-matched in age and clinical condition. Seroconversion in healthy controls ranged from 96 to 100% (supplementary fig. 9).

Of all studies searched, 14 reported the development of neutralizing antibodies against SARS-CoV-2 (1174 participants with HM). Response rate ranged across studies from 21.8 to 96.2% after the first measurement at least 14 days after the second vaccine (see supplementary table 7).

##### Cellular response

There were 11 studies reporting vaccine-induced T-cell responses in patients with HM. To evaluate such response, different assays were used across the studies and T-cell immunity was analyzed by intracellular cytokine staining and flow cytometry, measurement of secreted interferon-gamma (IFN-γ) by the ELISpot technique, or a whole-blood platform, and by measurement of secreted IFN-γ/Interleukin (IL)-2 by the FluoroSpot technique. T-cell response rates ranged from 26.5 to 85.9% in all studies (⊕⊕⊖⊖ low certainty of the evidence) (see Fig. 2F). Two studies providing data for HSCT patients only, reported the lowest response rates of 27% and 29%, respectively [41, 42]. Five studies did not further specify subgroups of hematological patients, three studies reported on multiple myeloma patients only [43–45], and the remaining study included various types of lymphoma [46]. The timepoint of T-cell measurement ranged between 14 days and 6 weeks after full primary immunization (two vaccines). Four studies further specified their results on the type of treatment with very small sample sizes in consequence. Yet, patients with anti-CD20 therapy within 6 months from vaccination were shown to have robust T-cell responses in 2/2 studies (100% response [*n* = 4] [47] and 75% [*n* = 33] [48]). In contrast, treatment with corticosteroids was associated with poor T-cell response [46].

A majority of studies (6/11) reported discordant immune responses with positive T-cell responses in patients lacking serological responses ranging from 19.7 to 74%. Aleman and colleagues performed in-depth analyses of such discordant responses and reported those T cells to be monofunctional and lower in their extent compared to T-cell responses in seropositive patients [43].

#### Safety

We identified six studies (853 participants with HM) reporting on adverse events (AEs). Across studies 0–50.9% of participants experienced at least one event (⊕⊕⊖⊖ low certainty of the evidence) (see Fig. 2G). Three studies did not report the observation period [28, 49, 50]. In addition, 17 studies reported local and/or systemic reactions or AEs of special interest for their study; an overview of reported events can be found in Supplementary Table 8. We identified three studies (208 participants with HM) reporting on vaccine-induced immune thrombotic thrombocytopenia (VITT). Across studies, 0–2.5% of participants experienced at least one event. One study did not report the observation period [37], one study observed participants for 49 days from the first dose[27], and one study for 2 weeks after the second dose [31]. Three studies (761 participants with HM) reported anaphylactic or systemic allergic reactions, 0–1.3% of participants experienced at least one event. Two studies (226 participants with HM) reporting the progress of the underlying HM were identified and 2.5–5.5% of participants reported experiencing progression of their underlying disease. Participants were followed for up to 1 month after the second vaccine dose; however, we did not identify any information on whether the disease progression was likely related or unrelated to the vaccination. We did not identify any studies reporting any events of myocarditis or termination of anti-cancer treatment due to vaccination.

Four studies (901 participants with HM) reported serious adverse events (SAEs). Across studies, 0–7.5% of participants experienced at least one event (⊕⊕⊖⊖ low certainty of the evidence) (see Fig. 2G). SAEs were assessed for at least 7 and up to 49 days following vaccination.

## Discussion

We here report the results of a systematic review assessing the effectiveness, immunogenicity, and safety of COVID-19 vaccines in patients with HM. Clinical outcomes assessing vaccine effectiveness were generally only occasionally and not systematically reported and further limited by short observational periods. Thus, a firm appraisal of the protective effect of COVID-19 vaccines on these highly patient-relevant outcomes is to date not possible. With regard to immunogenicity, we found heterogeneous vaccine-induced seroconversion rates and cellular immunity in HM patients, which was lower than reported for healthy participants in all studies. The highest humoral response rates were reported for myeloproliferative diseases and the lowest in CLL. Poor immunogenicity by COVID-19 vaccines in patients with an impaired B-cell axis is in line with other vaccines showing reduced efficacy in this population [51–55].

The detrimental effect of B-cell depleting treatment, especially when administered within 12 months from vaccine application, is concerning beyond the population of HM patients studied herein since these drugs are widely used beyond HM [56, 57]. Interestingly, initiating B-cell depleting treatment shortly after vaccination does not seem to relevantly impair humoral vaccine response [58]. The relatively low humoral response to vaccination in patients undergoing CAR-T-cell treatment compared to HSCT recipients could be attributable to the lymphodepleting chemotherapy and B-cell targeting constructs. Additionally, a historic effect, with HSCT recipients potentially recovered from treatment effects, appears plausible [59]. Due to different assays used and lacking international standards, a pooled outcome analysis of antibody titres was infeasible. However, it is generally questionable whether the serologic response can serve as reliable CoP in a population with an impaired B-cell axis.

In contrast to the serologic response to COVID-19 vaccines, insights into cellular vaccine response are still scarce. In the light of impaired serologic response in HM, SARS-CoV-2 specific T cells may be crucial in this population. Of 57 studies included, only 11 reported vaccine-induced T-cell response. Except for patients after allogeneic HSCT, robust T-cell responses were reported in HM patients across the studies included. Especially those receiving anti-CD20 treatment responded well on the T-cell level highlighting the protective capacities of vaccination also in patients with treatment-associated B cells [60]. Studies reporting on discordant immune responses show that at least 20% of patients show a T-cell response in the absence of seroconversion. These results are reassuring for those patients failing serologic response but question the role of antibodies as the sole correlate of protection. Caution is warranted; however, since most studies used different assays, different thresholds and usually lacked control groups. Further, the kinetics of T cells after COVID-19 vaccination in the immunocompromised remain sparsely investigated, and therefore, the ideal timepoint for T-cell measurement is yet to be determined.

With regard to safety, AE assessment and reporting were not performed systematically in most studies. However, reported events herein are largely consistent with the safety profile observed in clinical trials and real-world evidence of the general population [61–63]. AEs of special interest as identified and prioritized in the conducted patient workshop were rarely or never reported.

Given the rapid changes in the epidemiological situation and turnover of research output in the SARS-CoV-2 / COVID-19 field, it is nearly impossible to stay up to date with a methodologically sound and systematic approach. The problem of waning immunity was identified only in the last months and optimization schedules are widely investigated and partially already applied through an additional booster dose. A recent systematic review with meta-regression provides evidence of a decrease in vaccine effectiveness after full primary immunization against pre-omicron SARS-CoV-2 variant infection by 21% (95% CI 13.9–29.8) in the general population [64]. By the time of our search, we only identified a small number of studies investigating a third vaccine dose in HM patients. Interestingly, a more recent study reported successfully seroconversion after a third mRNA vaccine dose in 59% of 75 patients that had received cellular therapies or bispecific antibodies and failed the primary two-dose vaccine scheme [65]. This and other emerging studies highlight the potential of booster vaccinations and several international committees on immunization already recommend a fourth vaccine dose for immunologically vulnerable patient groups [66, 67].

None of the identified studies investigated vaccine effectiveness against the omicron VOC (BA.1, BA.2). First studies investigating neutralizing effectiveness against omicron in cancer patients suggest a higher immune evasive capacity; in individuals with HM also after receiving the third dose [68]. Even though the evidence synthesized here does not assess evidence on variant-specific vaccine effectiveness or effectiveness of (repeated) boosters, it provides a thorough summary of the capacity to develop a humoral and/or cellular response following vaccination for individuals with different HM stratified by disease entity and treatments received. Taken together, we identified growing evidence for humoral COVID-19 vaccine-induced humoral response, but scarce data for a cellular response, and even less evidence for clinical vaccine effectiveness and safety in HM patients. To date, patients with MM or CLL and those receiving B-cell depleting therapies are the best-described patient groups with regard to humoral response. In contrast, data for patients with other diseases such as acute leukemias or aggressive lymphoma are still largely lacking. As highlighted by the evidence-gap maps (Fig. 3) a large evidence gap remains for most HM patients and underlying treatment and/or disease status, while data on the cellular immune response is particularly scarce.

Overall, our systematic review highlights the generally poor immune response of people with HM to COVID-19. In light of easing or ending strategies aimed to reduce virus transmission for the general population, the flux in prevalent SARS-CoV-2 variants and entailing high incidence rates pose major risks to this vulnerable group. For high-risk individuals who respond poorly to current COVID-19 vaccination regimens, new approaches to prevent infections and severe or prolonged disease courses are urgently needed.

Further research should therefore evaluate improved booster immunization strategies including heterologous vaccinations for immunocompromised individuals, such as the herein reported group of HM patients. By defining standards for reporting vaccine efficacy on the humoral and cellular level and enforcing the adequate reporting of clinical outcomes, international boards of experts could guide future research to enhance cross-study comparability and maximize the evidence and benefit generated.

### Reporting summary

Further information on research design is available in the Nature Research Reporting Summary linked to this article.

## Supplementary information

Reporting Summary

Supplementary Material_for publication

Supplementary tables 4_5_and_6

supplementary figure 1

supplementary figure 2

supplementary figure 3

supplementary figure 4

supplementary figure 5

supplementary figure 6

supplementary figure 7

supplementary figure 8

supplementary figure 9

## Data Availability

The datasets generated during and/or analyzed during the current study are available from the corresponding author on reasonable request.
