# Supplementary material for: Effectiveness, immunogenicity, and safety of COVID-19 vaccines for individuals with hematological malignancies: a systematic review
Source: Blood Cancer J. 2022 May 31;12(5):86. doi: 10.1038/s41408-022-00684-8 (PMC9152308; doi:10.1038/s41408-022-00684-8)
Supplement: Supplementary file 2 — Supplementary Material_for publication [file 41408_2022_684_MOESM2_ESM.docx]

**Supplementary Material**

**Content**

[Methods 1](#_Toc103286191)

[**Search strategies** 1](#_Toc103286192)

[**Cochrane COVID-19 Study Register** 1](#_Toc103286193)

[**Web of Science (Core Collection) – Science Citation Index und Emerging Sources Citation Index** 2](#_Toc103286194)

[**WHO COVID 19 Global literature on coronavirus disease** 4](#_Toc103286195)

[**Risk of bias** 1](#_Toc103286196)

[**Changes between protocol and review** 2](#_Toc103286197)

[**Tables** 3](#_Toc103286198)

[Study characteristics 3](#_Toc103286199)

[**Effectiveness, Immunogenicity and Safety of COVID-19 Vaccines** 4](#_Toc103286200)

[List of supplementary figures 11](#_Toc103286201)

[References 12](#_Toc103286202)

# Methods

## **Search strategies**

### **Cochrane COVID-19 Study Register**

Search I

cancer* or neoplas* or malignan* or tumor* or tumour* or sarcom* or lymphom* or leukem* or leukaem* or hodgkin* or myelom* or myelodysplastic* or carcinoma* or onco* or haemato* or hemato* or chemotherap* or "chemo-therapy" or "cell transplantation" or "cell transplant" or "stem cell" or allogen* or autolog* or autograft* or allograft* or "bone marrow" or ASCT* or ABMT* or PBPC* or PBSCT* or PSCT* or BMT* or SCT* or HCT* or HSCT* or "anti-cancer" or anticancer* or antitumor* or antitumour* or "anti-tumor" or "anti-tumour" or antineoplastic* or "B-cell directed" or "B-cell depletion" or "B-cell disruption" or "B-Cell depleted" or "B-Cell depleting" or "B-cell directed therapy" or "CAR-T" or "T cell therapy" or "CART" or "cellular therapy" or "cell therapy"

AND

vaccin*

AND

COVID or COVID19 or "SARS-CoV-2" or "SARS-CoV2" or SARSCoV2 or "SARSCoV-2" or "SARS coronavirus 2" or "2019 nCoV" or "2019nCoV" or "2019-novel CoV" or "nCov 2019" or "nCov 19" or "severe acute respiratory syndrome coronavirus 2" or "novel coronavirus disease" or "novel corona virus disease" or "corona virus disease 2019" or "coronavirus disease 2019" or "novel coronavirus pneumonia" or "novel corona virus pneumonia" or "severe acute respiratory syndrome coronavirus 2"

Search *II*

cancer* or neoplas* or malignan* or tumor* or tumour* or sarcom* or lymphom* or leukem* or leukaem* or hodgkin* or myelom* or myelodysplastic* or carcinoma* or onco* or haemato* or hemato* or chemotherap* or "chemo-therapy" or "cell transplantation" or "cell transplant" or "stem cell" or allogen* or autolog* or autograft* or allograft* or "bone marrow" or ASCT* or ABMT* or PBPC* or PBSCT* or PSCT* or BMT* or SCT* or HCT* or HSCT* or "anti-cancer" or anticancer* or antitumor* or antitumour* or "anti-tumor" or "anti-tumour" or antineoplastic* or "B-cell directed" or "B-cell depletion" or "B-cell disruption" or "B-Cell depleted" or "B-Cell depleting" or "B-cell directed therapy" or "CAR-T" or "T cell therapy" or "CART" or "cellular therapy" or "cell therapy"

AND

biontech* or pfizer* or corminaty* or comirnaty* or BNT162* or "BNT 162" or "bnt 162b2" or tozinameran* or moderna* or spikevax* or 1273* or mRNA1273* or "TAK-919" or "CX-024414" or CX024414* or astrazeneca* or vaxzevria* or AZD1222* or covishield* or ChAdOx* or Janssen* or "JNJ-78436735" or JNJ78436735* or VAC31518* or "VAC-31518" or "Johnson COVID-19" or "Johnson COVID19" or Ad26* or Ad5* or Sputnik* or rAd26* or rAd5* or gamaleya* or "Gam-COVID-Vac" or "recombinant adenovirus type" or "adenovirus vector" or "combined vector" or BBIBP* or sinopharm* or sinovac* or PiCoVac* or Coronavac* or "heterologous boost" or "heterologous booster" or "homologous boost" or "homologous booster" or "post-boost" or "post-booster" or "boost schedule" or "boost schedules" or "bost dose" or "booster dose" or "after boost" or "after booster" or "variant boost" or "variant booster" or "nonvariant boost" or "nonvariant booster" or "non-variant boost" or "non-variant booster" or "delayed boost" or "delayed booster" or boosted or "prime boost" or "prime booster" or "boost regimen" or "three dosis" or "three doses" or "third dosis" or "third doses" or "third dose"

**Filter according to**

Study Characteristics: Study Design

- Case Series/Case Control/Cohort

- Parallel/Crossover

- Cross-Sectional

- Single Arm/Controlled Before After

- Other

- Unclear

- Time series

### **Web of Science (Core Collection) – Science Citation Index und Emerging Sources Citation Index**

• Science Citation Index Expanded (1945-present)

• Emerging Sources Citation Index (2015-present)

• Limit to 01-03-2020 – present

#1 (TI=(COVID OR COVID19 OR "SARS-CoV-2" OR "SARS-CoV2" OR SARSCoV2 OR "SARSCoV-2" OR "SARS coronavirus 2" OR "2019 nCoV" OR "2019nCoV" OR "2019-novel CoV" OR "nCov 2019" OR "nCov 19" OR "severe acute respiratory syndrome coronavirus 2" OR "novel coronavirus disease" OR "novel corona virus disease" OR "corona virus disease 2019" OR "coronavirus disease 2019" OR "novel coronavirus pneumonia" OR "novel corona virus pneumonia" OR "severe acute respiratory syndrome coronavirus 2")) OR AB=(COVID OR COVID19 OR "SARS-CoV-2" OR "SARS-CoV2" OR SARSCoV2 OR "SARSCoV-2" OR "SARS coronavirus 2" OR "2019 nCoV" OR "2019nCoV" OR "2019-novel CoV" OR "nCov 2019" OR "nCov 19" OR "severe acute respiratory syndrome coronavirus 2" OR "novel coronavirus disease" OR "novel corona virus disease" OR "corona virus disease 2019" OR "coronavirus disease 2019" OR "novel coronavirus pneumonia" OR "novel corona virus pneumonia" OR "severe acute respiratory syndrome coronavirus 2")

#2 (TI=(biontech* or pfizer* or corminaty* or comirnaty* or BNT162* or "BNT 162" or "bnt 162b2" or tozinameran* or moderna* or spikevax* or 1273* or mRNA1273* or "TAK-919" or "CX-024414" or CX024414* or astrazeneca* or vaxzevria* or AZD1222* or covishield* or ChAdOx* or Janssen or "JNJ-78436735" or JNJ78436735* or "Johnson COVID-19" or "Johnson COVID19" or Ad26* or Ad5* or VAC31518* or Sputnik* or rAd26* or rAd5* or gamaleya* or "Gam-COVID-Vac" or "recombinant adenovirus type" or "adenovirus vector" or "combined vector" or BBIBP* or sinopharm* or sinovac* or PiCoVac* or Coronavac*)) OR AB=(biontech* or pfizer* or corminaty* or comirnaty* or BNT162* or "BNT 162" or "bnt 162b2" or tozinameran* or moderna* or spikevax* or 1273* or mRNA1273* or "TAK-919" or "CX-024414" or CX024414* or astrazeneca* or vaxzevria* or AZD1222* or covishield* or ChAdOx* or Janssen or "JNJ-78436735" or JNJ78436735* or "Johnson COVID-19" or "Johnson COVID19" or Ad26* or Ad5* or VAC31518* or Sputnik* or rAd26* or rAd5* or gamaleya* or "Gam-COVID-Vac" or "recombinant adenovirus type" or "adenovirus vector" or "combined vector" or BBIBP* or sinopharm* or sinovac* or PiCoVac* or Coronavac*)

#3 (TI=(boost*)) OR AB=(boost*)

#4 (TI=("three dosis" OR "three doses" OR "third dosis" OR "third doses" OR "third dose")) OR AB=("three dosis" OR "three doses" OR "third dosis" OR "third doses" OR "third dose")

#5 (TI=((vaccin* NEAR/5 (COVID OR COVID19 OR "SARS-CoV-2" OR "SARS-CoV2" OR SARSCoV2 OR "SARSCoV-2" OR "SARS coronavirus 2" OR "2019 nCoV" OR "2019nCoV" OR "2019-novel CoV" OR "nCov 2019" OR "nCov 19" OR "severe acute respiratory syndrome coronavirus 2" OR "novel coronavirus disease" OR "novel corona virus disease" OR "corona virus disease 2019" OR "coronavirus disease 2019" OR "novel coronavirus pneumonia" OR "novel corona virus pneumonia" OR "severe acute respiratory syndrome coronavirus 2")))) OR AB=((vaccin* NEAR/5 (COVID OR COVID19 OR "SARS-CoV-2" OR "SARS-CoV2" OR SARSCoV2 OR "SARSCoV-2" OR "SARS coronavirus 2" OR "2019 nCoV" OR "2019nCoV" OR "2019-novel CoV" OR "nCov 2019" OR "nCov 19" OR "severe acute respiratory syndrome coronavirus 2" OR "novel coronavirus disease" OR "novel corona virus disease" OR "corona virus disease 2019" OR "coronavirus disease 2019" OR "novel coronavirus pneumonia" OR "novel corona virus pneumonia" OR "severe acute respiratory syndrome coronavirus 2")))

#6 1 AND (#2 OR #3 OR #4)

#7 #5 OR #6

#8 (TI=(cancer* OR neoplas* OR malignan* OR tumor* OR tumour* OR sarcom* OR lymphom* OR leukem* OR leukaem* OR hodgkin* OR myelom* OR myelodysplastic* OR carcinoma* OR onco* OR haemato* OR hemato* OR chemotherap* OR "chemo-therapy" OR "cell transplantation" OR "cell transplant" OR "stem cell" OR allogen* OR autolog* OR autograft* OR allograft* OR "bone marrow" OR ASCT* OR ABMT* OR PBPC* OR PBSCT* OR PSCT* OR BMT* OR SCT* OR HCT* OR HSCT* OR "anti-cancer" OR anticancer* OR antitumor* OR antitumour* OR "anti-tumor" OR "anti-tumour" OR antineoplastic* OR "B-cell directed" OR "B-cell depletion" OR "B-cell disruption" OR "B-Cell depleted" OR "B-Cell depleting" OR "B-cell directed therapy" OR "CAR-T" OR "T cell therapy" OR "CART" OR "cellular therapy" OR "cell therapy")) OR AB=(cancer* OR neoplas* OR malignan* OR tumor* OR tumour* OR sarcom* OR lymphom* OR leukem* OR leukaem* OR hodgkin* OR myelom* OR myelodysplastic* OR carcinoma* OR onco* OR haemato* OR hemato* OR chemotherap* OR "chemo-therapy" OR "cell transplantation" OR "cell transplant" OR "stem cell" OR allogen* OR autolog* OR autograft* OR allograft* OR "bone marrow" OR ASCT* OR ABMT* OR PBPC* OR PBSCT* OR PSCT* OR BMT* OR SCT* OR HCT* OR HSCT* OR "anti-cancer" OR anticancer* OR antitumor* OR antitumour* OR "anti-tumor" OR "anti-tumour" OR antineoplastic* OR "B-cell directed" OR "B-cell depletion" OR "B-cell disruption" OR "B-Cell depleted" OR "B-Cell depleting" OR "B-cell directed therapy" OR "CAR-T" OR "T cell therapy" OR "CART" OR "cellular therapy" OR "cell therapy")

#9 #7 AND #8

### **WHO COVID 19 Global literature on coronavirus disease**

Search I

cancer* or neoplas* or malignan* or tumor* or tumour* or sarcom* or lymphom* or leukem* or leukaem* or hodgkin* or myelom* or myelodysplastic* or carcinoma* or onco* or haemato* or hemato* or chemotherap* or "chemo-therapy" or "cell transplantation" or "cell transplant" or "stem cell" or allogen* or autolog* or autograft* or allograft* or "bone marrow" or ASCT* or ABMT* or PBPC* or PBSCT* or PSCT* or BMT* or SCT* or HCT* or HSCT* or "anti-cancer" or anticancer* or antitumor* or antitumour* or "anti-tumor" or "anti-tumour" or antineoplastic* or "B-cell directed" or "B-cell depletion" or "B-cell disruption" or "B-Cell depleted" or "B-Cell depleting" or "B-cell directed therapy" or "CAR-T" or "T cell therapy" or "CART" or "cellular therapy" or "cell therapy"

AND

vaccin*

AND

COVID or COVID19 or "SARS-CoV-2" or "SARS-CoV2" or SARSCoV2 or "SARSCoV-2" or "SARS coronavirus 2" or "2019 nCoV" or "2019nCoV" or "2019-novel CoV" or "nCov 2019" or "nCov 19" or "severe acute respiratory syndrome coronavirus 2" or "novel coronavirus disease" or "novel corona virus disease" or "corona virus disease 2019" or "coronavirus disease 2019" or "novel coronavirus pneumonia" or "novel corona virus pneumonia" or "severe acute respiratory syndrome coronavirus 2"

search II

cancer* or neoplas* or malignan* or tumor* or tumour* or sarcom* or lymphom* or leukem* or leukaem* or hodgkin* or myelom* or myelodysplastic* or carcinoma* or onco* or haemato* or hemato* or chemotherap* or "chemo-therapy" or "cell transplantation" or "cell transplant" or "stem cell" or allogen* or autolog* or autograft* or allograft* or "bone marrow" or ASCT* or ABMT* or PBPC* or PBSCT* or PSCT* or BMT* or SCT* or HCT* or HSCT* or "anti-cancer" or anticancer* or antitumor* or antitumour* or "anti-tumor" or "anti-tumour" or antineoplastic* or "B-cell directed" or "B-cell depletion" or "B-cell disruption" or "B-Cell depleted" or "B-Cell depleting" or "B-cell directed therapy" or "CAR-T" or "T cell therapy" or "CART" or "cellular therapy" or "cell therapy"

AND

biontech* or pfizer* or corminaty* or comirnaty* or BNT162* or "BNT 162" or "bnt 162b2" or tozinameran* or moderna* or spikevax* or 1273* or mRNA1273* or "TAK-919" or "CX-024414" or CX024414* or astrazeneca* or vaxzevria* or AZD1222* or covishield* or ChAdOx* or Janssen* or "JNJ-78436735" or JNJ78436735* or VAC31518* or "VAC-31518" or "Johnson COVID-19" or "Johnson COVID19" or Ad26* or Ad5* or Sputnik* or rAd26* or rAd5* or gamaleya* or "Gam-COVID-Vac" or "recombinant adenovirus type" or "adenovirus vector" or "combined vector" or BBIBP* or sinopharm* or sinovac* or PiCoVac* or Coronavac* or "heterologous boost" or "heterologous booster" or "homologous boost" or "homologous booster" or "post-boost" or "post-booster" or "boost schedule" or "boost schedules" or "bost dose" or "booster dose" or "after boost" or "after booster" or "variant boost" or "variant booster" or "nonvariant boost" or "nonvariant booster" or "non-variant boost" or "non-variant booster" or "delayed boost" or "delayed booster" or boosted or "prime boost" or "prime booster" or "boost regimen" or "three dosis" or "three doses" or "third dosis" or "third doses" or "third dose"

## **Risk of bias**

Due to the change of focus of our review question from intervention comparison to overall outcomes for haematological patients after SARS-CoV-2 vaccines, the tools we originally defined for our question, the Cochrane RoB-2 tool and ROBINS-I (1, 2), which are tailored to assess intervention comparisons, were not applicable. Currently, no one recommended tool to systematically assess the risk of bias and applicability in overall prognosis or single arm studies; we therefore used a tool which is currently in its first phase of development by members of the Cochrane Prognosis Methods Group and Cochrane Haematology, the Risk of Bias in Overall Prognosis Studies (ROB-OPS) tool.

The draft was based on existing questions from tools such as the Prediction model Risk Of Bias Assessment Tool (PROBAST)(3), the Quality In Prognosis Studies tool (QUIPS)(4), Risk Of Bias In Non-randomised Studies - of Interventions (ROBINS-I) (1), and 2 (RoB-2)(2) tools that were evaluated for eligibility for this type of studies. It consists of two parts, a section for applicability and one for risk of bias, and covers the domains participants, context and outcome for applicability, and the domains participants, outcome, analysis and selective reporting for the risk of bias section (see Table 1). Each domain contains one or more signalling questions and rating options to guide to either “yes”, “probably yes”, “probably no”, “no” or “not sufficient information”.

**Table 1: Risk of Bias in Overall Prognosis Studies (ROB-OPS) tool, Version 0.1**

| Domain | Signalling question |
| --- | --- |
| 1. Participants | 1.1 Was the method of participant recruitment appropriate? |
|  | 1.2 Were all inclusions and exclusions appropriate? |
| 2. Outcome | 2.1 Was the outcome of interest defined appropriately? |
|  | 2.2 Was the used outcome measure appropriate? |
|  | 2.3 Was the duration of follow-up appropriate? |
|  | 2.4 Was the outcome determined without knowledge of relevant baseline characteristics? |
| 3. Analysis | 3.1 Were outcome data available for all included participants or likely to be unrelated to the outcome value? |
|  | 3.2 Were missing outcome data handled appropriately? |
|  | 3.3 Was the statistical method for estimating the overall prognosis appropriate? |
|  | 3.4 Were complexities in the analysed data handled appropriately? |
| 4. selective reporting | 4.1 Is the reported outcome likely to be unselected? |

We assessed applicability and risk of bias per study and per outcome groups (i.e., efficacy, immunogenicity and safety). Each domain received an overall rating of either low, moderate or high rating for risk of bias. Based on the domain-rating for each study and outcome group, an overall rating for the outcome group per study was created as follows:

- Low risk of bias: no domain rated as high or moderate risk of bias per study and outcome group
- Moderate risk of bias: at least one rating as moderate risk of bias per study and outcome group, and none with high risk. For those studies with more than one outcome rated per outcome group, the overall risk of bias was rated for each study outcome separately.
- High risk of bias: at least one domain rated as high risk of bias per study and outcome group, or at least two domains rated as moderate risk of bias. For those studies with more than one outcome rated per outcome group, the overall risk of bias was rated for each study outcome separately

## **Changes between protocol and review**

During the review process, several changes were decided upon in group discussions by the complete author team (Table 2).

**Table 2: Changes between the protocol and the review**

| **Topic** | **Protocol** | **Review** | **Reasoning** |
| --- | --- | --- | --- |
| Review scope/question | Intervention review with focus on vaccine comparison | Overall prognosis review type with focus on overall frequencies | Due to a lack of randomised controlled or prospective controlled studies with vaccine comparisons, we decided to shift our focus and summarise outcomes for all HM and subgroups, in addition to a comparison of vaccine responses as far as it was possible |
| Eligibility criteria | We planned to include conference abstracts. | Conference abstracts excluded | Limited information provided by abstracts, both regarding data and information for risk of bias assessment. |
| Procedures | Data extraction in duplicate | Data extraction by one author and data check by at least a second author | Resource-limitation |
|  | Data synthesis for RCTs, non-randomised controlled studies and single-arm/ prospective cohort studies | Data synthesis only possible for single-arm studies | Only single-arm/prospective cohort studies identified. |
|  | Data synthesis via RevMan Web | Data synthesis via software R | Change of focus in review question required more flexible data processing and recalculation of confidence intervals. |
| Risk of bias assessment | Cochrane Risk of Bias 2 tool or ROBINS-I tool | Self-developed tool “RoB-OPS” | Due to the shift of focus on non-controlled studies, RoB-2 and ROBINS-I were not applicable anymore. A preliminary version of RoB-OPS, a currently ongoing project, was used instead. |
| Subgroups | Planned subgroup: Vaccination scheme (duration between vaccinations, heterologous vs. homogenous, time interval to booster shot | We did not perform subgroup analysis according to vaccination scheme | Time frame between vaccinations were relatively homogeneous according to standard protocol; nearly no booster studies identified up to search |

# **Tables**

## Study characteristics

**Table 3. List of excluded studies**

| **Excluded studies** | **Reason for exclusion** |
| --- | --- |
| (5-8) | Mixed cohort and data for fully vaccinated HM not separately reported |
| (9-21) | First vaccine dose |
| (22-28) | Fulltext not retrievable |
| (29-33) | Abstract publication |
| (34-36) | Small sample size (<20 participants) |
| (37, 38) | Ineligible intervention |
| (39-45) | Ineligible outcomes |
| (46-90) | Ineligible population |
| (91-98) | Ineligible publication type |
| (99-132) | Ineligible study design |

**Table 4. Overview of ongoing studies**

Please refer to provided Excel spreadsheet.

**Table 5. Characteristics of included studies**

Please refer to provided Excel spreadsheet.

**Table 6. Laboratory tests**

Please refer to provided Excel spreadsheet.

## **Effectiveness, Immunogenicity and Safety of COVID-19 Vaccines**

**Table 7. Outcome summary**

| **Outcome** | **Range of reported effect rates** | **Number of participants (studies)** |
| --- | --- | --- |
| *Clinical outcomes* | | |
| SARS-CoV-2 infection | 0-11·9% | 3277 (19) |
| Time to infection | 16-23% | 72 (1) |
| Symptomatic COVID-19 | 0- 2·7% | 1484 (13) |
| Hospital admission | 0-2·8% | 382 (6) |
| ICU admission | 0% | 72 (1) |
| Mortality | 0-0·5% | 1228 (6) |
| Transmissibility | NA | no studies identified |
| Quality of life | NA | no studies identified |
| *Immunity parameters* | | |
| Antibody response | 38·1-99·1% | 7393 (48) |
| Neutralizing antibody response | 21·8-96·2% | 1174 (14) |
| T-cell response | 26·5-85·9% | 970 (11) |
| B-cell response | NA | no studies identified |
| Waning of immunity | NA | no studies identified |
| *Safety aspects* | | |
| Adverse events | 0-50·9% | 853 (6)* |
| VITT | 0-2·5% | 208 (3) |
| Anaphylactic or systemic allergic reactions | 0-1·3% | 761 (3) |
| Progress or relapse of HM | 2·5-5·5% | 226 (2) |
| Myocarditis | NA | no studies identified |
| Termination of anti-cancer treatment | NA | no studies identified |
| Serious adverse events | 0-7·5% | 901 (4) |

*several studies report local and/or systemic reactions, or AEs of special interest for the study; an overview can be found in the appendix

Abbreviations:

ICU, intensive care unit; VITT, vaccine-induced immune thrombotic thrombocytopenia; HM, haematological malignancy;

**Table 8. Overview of any reported adverse events**

| **Study Name** | **Outcome definition** | **Time of Follow-up** | **Number of participants with event** |
| --- | --- | --- | --- |
| Ram 2021(192) | non-hematologic vaccine-reported adverse events | after second dose | 18/80 |
|  | possibly vaccine-related hematologic adverse events | after second dose | 8/80 |
|  | non-hematologic adverse events grade 3 or 4 | after second dose | 0/80 |
|  | Graft-versus host disease (GVHD) exacerbation | after second dose | 3/80 |
| Eifer 2021 | Axillary lymph node PET/CT uptake | 1-34 days (IQR 6-21) between vaccination and imaging | 24/75 |
| Gavriatopoulou 2021 | mild reactions (pain at the site of the injection, erythema, and/or swelling) after BNT162b2 | after second dose | 34/90 |
|  | mild systemic adverse reactions (fatigue, fever, lymphadenopathy,muscle pain, arthargias, and headache) after mRNA-1273 | after second dose | 22/90 |
| Terpos 2021 | systemic adverse reactions which included fatigue, fever, and lymphadenopathy | after second dose | 8/132 |
|  | systemic adverse reactions (grade 1 or 2) after BNT162b2 | after second dose | 45/215 |
|  | local reactions after AZD1222 | after first dose | 20/61 |
|  | mild reactions after BNT162b2 | after second dose | 68/215 |
| Maneikis 2021 | AEs mild/grade I | 7 days after second dose | Only reported in figure |
|  | AEs moderate/grade II | 7 days after second dose | Only reported in figure |
|  | AEs severe/grade III | 7 days after second dose | Only reported in figure |
|  | AEs potentially life-threatening/grade IIII | 7 days after second dose | 0/575 |
| Benjamini 2021 | grade 1 side effects in patients with present immune response | on day of serologic test after second vaccine (median 19 days, range 12-53) | 62/138 |
|  | grade 1 side effects in patients with absent immune response | on day of serologic test (14-21 days after second vaccine (median 19 days (range 12-53))) | 76/138 |
|  | grade 1 pain in patients with present immune response | on day of serologic test (14-21 days after second vaccine (median 19 days (range 12-53))) | 38/91 |
|  | grade 1 pain in patients with absent immune response | on day of serologic test (14-21 days after second vaccine (median 19 days (range 12-53))) | 53/91 |
| Avivi, Herishanu 2021 | any AEs | within 7 days after first dose | 64/167 |
|  | any AEs | within 7 days after second dose | 79/167 |
|  | only local AEs | within 7 days after first dose | 43/167 |
|  | only local AEs | within 7 days after second dose | 40/167 |
|  | systemic AEs | within 7 days after first dose | 21/167 |
|  | systemic AEs | within 7 days after second dose | 39/167 |
| Perry 2021 | Adverse events | within 7 days after each vaccination | 60/118 |
| Canti 2021 | pain at the site of vaccination | during 49 days after first vaccine | 34/40 |
|  | fatigue | during 49 days after first vaccine | 16/40 |
|  | headache | during 49 days after first vaccine | 12/40 |
|  | myalgia | during 49 days after first vaccine | 11/40 |
|  | chills | during 49 days after first vaccine | 6/40 |
|  | rash | during 49 days after first vaccine | 1/40 |
|  | exacerbation of chronic obstructive pulmonary disease | during 49 days after first vaccine | 1/40 |
| Cattaneo 2021 | adverse events | NR | 0/62 |
| Molica 2021 | systemic adverse events | after first dose | 8/70 |
|  | systemic adverse events | after second dose | 9/70 |
| Yeshurun 2021 | grade 4 steroid-refractory immune thrombocytopenia 2 | 2 weeks after first vaccine | 1/106 |
|  | worsening of GVHD-related symptoms | within days following the first, second or both vaccines | 7/106 |
| Pinana 2021 | self-reported mild AEs | NR | 35/397 |
| Rahav 2021 | Local events (pain, tenderness, erythema, induration, lymphadenopathy) | 4 weeks after first dose | 11/111 (HSCT) |
|  | Local events (pain, tenderness, erythema, induration, lymphadenopathy) | 4 weeks after second dose | 13/111 (HSCT) |
|  | Systemic events (fatigue, headache, fever, myalgia, paresthesia, other) | 4 weeks after first dose | 6/111 (HSCT) |
|  | Systemic events (fatigue, headache, fever, myalgia, paresthesia, other) | 4 weeks after second vaccination | 15/111 (HSCT) |
|  | Local events (pain, tenderness, erythema, induration, lymphadenopathy) | 4 weeks after first dose | 68/188 (CLL/NHL) |
|  | Local events (pain, tenderness, erythema, induration, lymphadenopathy) | 4 weeks after second dose | 73/188 (CLL/NHL) |
|  | Systemic events (fatigue, headache, fever, myalgia, paresthesia, other) | 4 weeks after first dose | 15/188 (CLL/NHL) |
|  | Systemic events (fatigue, headache, fever, myalgia, paresthesia, other) | 4 weeks after second vaccination | 22/188 (CLL/NHL) |
|  | GVHD | 4 weeks after second vaccination | 0/529 |
| Figueriedo 2021 | adverse reactions (self-administered symptom questionnaire) | after second dose | 28/80 (HM) |
|  | adverse reactions (self-administered symptom questionnaire) | after second dose | 23/64 (B-cell targeted) |
| Reimann 2021 | Any AEs | up to 7 days after heterologous boost | 9/29 |
|  | Severe AEs | up to 7 days after heterologous boost | 2/29 |
|  | Pain at injection side | up to 7 days after heterologous boost | 2/29 |
|  | Fatigue | up to 7 days after heterologous boost | 7/29 |
|  | headache | up to 7 days after heterologous boost | 1/29 |
|  | Joint pain | up to 7 days after heterologous boost | 1/29 |
|  | fever | up to 7 days after heterologous boost | 1/29 |
| Sherman 2021 | injection site pain (all mild) | up to 7 days after first dose | 10/18 |
|  | headache (mild to moderate) | up to 7 days after first dose | 3/18 |
|  | fatigue (mild to moderate) | up to 7 days after first dose | 5/18 |
|  | gastrointestinal symptoms (mild to moderate) | up to 7 days after first dose | 2/18 |
|  | fever (any grade) | up to 7 days after first dose | 0/18 |
|  | myalgia (any grade) | up to 7 days after first dose | 0/18 |
|  | chills (any grade) | up to 7 days after first dose | 0/18 |
|  | injection site pain (mild to severe) | up to 7 days after second dose | 10/18 |
|  | headache (mild to severe) | up to 7 days after second dose | 4/18 |
|  | fatigue (mild to severe) | up to 7 days after second dose | 8/18 |
|  | gastrointestinal symptoms (mild to severe) | up to 7 days after second dose | 3/18 |
|  | fever (low grade) | up to 7 days after second dose | 2/18 |
|  | myalgias (mild to severe) | up to 7 days after second dose | 5/18 |
|  | chills (mild to severe) | up to 7 days after second dose | 3/18 |
| Maneikis 2021 | vomiting (grade I to II) | up to 7 days after first dose | 3/662 |
|  | diarrhea (grade I to III) | up to 7 days after first dose | 6/662 |
|  | fever (grade I) | up to 7 days after first dose | 6/662 |
|  | chills (grade I to II) | up to 7 days after first dose | 23/662 |
|  | fatigue (grade I to II) | up to 7 days after first dose | 49/662 |
|  | headache (grade I to II) | up to 7 days after first dose | 48/662 |
|  | muscle pain (grade I to III) | up to 7 days after first dose | 64/662 |
|  | joint pain (grade I to III) | up to 7 days after first dose | 15/662 |
|  | vomiting (grade I to II) | up to 7 days after second dose | 5/575 |
|  | diarrhea (grade I to II) | up to 7 days after second dose | 6/575 |
|  | fever (grade II to II) | up to 7 days after second dose | 24/575 |
|  | chills (grade I to III) | up to 7 days after second dose | 31/575 |
|  | fatigue (grade I to III) | up to 7 days after second dose | 72/575 |
|  | headache (grade I to III) | up to 7 days after second dose | 52/575 |
|  | muscle pain (grade I to III) | up to 7 days after second dose | 44/575 |
|  | joint pain (grade I to III) | up to 7 days after second dose | 24/575 |
| Shem-Tov 2021 | local AE after first vaccine (pain at injection site, erythaema, swelling) | "within 30 days after vaccination" | 15/152 |
|  | local AE after second vaccine | "within 30 days after vaccination" | 18/152 |
|  | systemic AE after first vaccine (fever, fatigue, headache, myalgia, chills, nausea/vomiting, paraesthesia) | "within 30 days after vaccination" | 8/152 |
|  | systemic AE after second vaccine | "within 30 days after vaccination" | 20/152 |
| Schiller 2021 | Vaccination-related local pain | during the first month after the second vaccine dose | 19/186 |
|  | Vaccination-related fever | during the first month after the second vaccine dose | 3/186 |
|  | Vaccination-related muscle pain | during the first month after the second vaccine dose | 5/186 |
| Cohen 2021 | Vaccine-associated hypermetabolic lymphadenopathy | NR | 43/137 |

Abbreviations: AE, adverse event; GvHD, graft-versus-host disease

# List of supplementary figures

Supplementary figure 1: Risk of bias assessments for clinical outcomes

Supplementary figure 2: Risk of bias assessments for immunogenicity outcomes

Supplementary figure 3: Risk of bias assessments for safety outcomes

Supplementary figure 4: Humoral response rate per type of disease

Supplementary figure 5: Humoral response rate per type of therapy

Supplementary figure 6: Humoral response rate after initial and extended follow-up

Supplementary figure 7: Humoral response rate after booster vaccination

Supplementary figure 8: Humoral response rate per vaccine type

Supplementary figure 9: Humoral response rate for HM patients and healthy controls

# References

1. Sterne JAC, Hernán MA, Reeves BC, Savović J, Berkman ND, Viswanathan M, et al. ROBINS-I: a tool for assessing risk of bias in non-randomised studies of interventions. BMJ. 2016;355:i4919.

2. Sterne JAC, Savović J, Page MJ, Elbers RG, Blencowe NS, Boutron I, et al. RoB 2: a revised tool for assessing risk of bias in randomised trials. BMJ. 2019;366:l4898.

3. Wolff RF, Moons KGM, Riley RD, Whiting PF, Westwood M, Collins GS, et al. PROBAST: A Tool to Assess the Risk of Bias and Applicability of Prediction Model Studies. (1539-3704 (Electronic)).

4. Hayden JA, van der Windt Da Fau - Cartwright JL, Cartwright Jl Fau - Côté P, Côté P Fau - Bombardier C, Bombardier C. Assessing bias in studies of prognostic factors. (1539-3704 (Electronic)).

5. Pagano L S-GJMFL-GALSIFGdSMDGF-RIvDJS. COVID-19 in vaccinated adult patients with hematological malignancies. Preliminary results from EPICOVIDEHA. Blood. 2021.

6. Le Bourgeois A C-BMGTPPGABMCCP. Safety and Antibody Response after 1 and 2 Doses of BNT162b2 mRNA Vaccine in Recipients of Allogeneic Hematopoietic Stem Cell Transplant. JAMA network open. 2021.

7. Mamez Ac PAGFPAUMFVDLM-LSMSDCC-RDEC. Antibody responses to SARS-CoV2 vaccination in allogeneic hematopoietic stem cell transplant recipients. Bone marrow transplantation. 2021.

8. Matkowska-Kocjan A O-LJCJKESFJLWMBDSLUM. The COVID-19 mRNA BNT163b2 Vaccine Was Well Tolerated and Highly Immunogenic in Young Adults in Long Follow-Up after Haematopoietic Stem Cell Transplantation. Vaccines. 2021;9(10).

9. Catherine D, Jessica C, Akiko K, Michael G, Judith G, Bruce R, et al. Impaired Humoral Immunity to SARS-CoV-2 Vaccination in Non-Hodgkin Lymphoma and CLL Patients. 2021.

10. Chevallier P C-BMLBAPPGABMCIBMDTLGSMPM. Safety and immunogenicity of a first dose of SARS-CoV-2 mRNA vaccine in allogeneic hematopoietic stem-cells recipients. Ejhaem. 2021.

11. Chowdhury O, Bruguier H, Mallett G, Sousos N, Crozier K, Allman C, et al. Impaired antibody response to COVID-19 vaccination in patients with chronic myeloid neoplasms. Br J Haematol.

12. Gastinne T, Le Bourgeois A, Coste-Burel M, Guillaume T, Peterlin P, Garnier A, et al. Safety and antibody response after one and/or two doses of BNT162b2 Anti-SARS-CoV-2 mRNA vaccine in patients treated by CAR T cells therapy. Br J Haematol.

13. Gavriatopoulou M TEKEBAGSN-SISADMPE-PEMMTIP. Low neutralizing antibody responses in WM, CLL and NHL patients after the first dose of the BNT162b2 and AZD1222 vaccine. Clinical and experimental medicine. 2021.

14. Ghandili S SMLMSZWJBHBCSMWKCLLB. Post-Vaccination Anti-SARS-CoV-2-Antibody Response in Patients with Multiple Myeloma Correlates with Low CD19+ B-Lymphocyte Count and Anti-CD38 Treatment. Cancers. 2021;13(15).

15. Guglielmelli P MAMLKSTZLPSRASMBMBARGMA. Impaired response to first SARS-CoV-2 dose vaccination in myeloproliferative neoplasm patients receiving ruxolitinib. American journal of hematology. 2021.

16. Harrington P DKJRDORALHPJSJGCLTMDDRS. Single dose of BNT162b2 mRNA vaccine against severe acute respiratory syndrome coronavirus-2 (SARS-CoV-2) induces neutralising antibody and polyfunctional T-cell responses in patients with chronic myeloid leukaemia. Br J Haematol. 2021.

17. Harrington P DKRDRALHPJSJGCLTMDDRSYE. Single dose of BNT162b2 mRNA vaccine against SARS-CoV2 induces neutralizing antibody and polyfunctional T-cell responses in patients with CML (preprint). Medrxiv. 2021:2021.04.15.21255482.

18. Ramasamy K SRJSVSTALJGNBJBSCGKCSDDM. COVID symptoms, testing, shielding impact on patient-reported outcomes and early vaccine responses in individuals with multiple myeloma. Br J Haematol. 2021.

19. Terpos E, Trougakos IP, Gavriatopoulou M, Papassotiriou I, Sklirou AD, Ntanasis-Stathopoulos I, et al. Low neutralizing antibody responses against SARS-CoV-2 in older patients with myeloma after the first BNT162b2 vaccine dose. Blood. 2021;137(26):3674-6.

20. Terpos E TIPGMPISADN-SIPEDFDKEDMA. Low Neutralizing Antibody Responses Against SARS-CoV-2 in Elderly Myeloma Patients After the First BNT162b2 Vaccine Dose. Blood. 2021(26).

21. Guglielmelli P, Mazzoni A, Maggi L, Kiros ST, Zammarchi L, Pilerci S, et al. Impaired response to first SARS-CoV-2 dose vaccination in myeloproliferative neoplasm patients receiving ruxolitinib. American Journal of Hematology.

22. Van Oekelen O GCRASSKBKFAAKKPVIStMTH. Highly variable SARS-CoV-2 spike antibody responses to two doses of COVID-19 RNA vaccination in patients with multiple myeloma. Cancer cell. 2021.

23. Southall JR. COVID-19 vaccine responses vary after treatment for lymphoid malignancies. HEM/ONC Today. 2021;22(16):2-.

24. Elisa R, Moraima J, Guillermo V, Isabel RC, Candela FN, Laura FM, et al. The Mrna-1273 Vaccine against Sars-Cov-2 Is Effective and Safe in Patients Undergoing Hematopoietic Progenitor Transplantation. Haematologica. 2021;106(10):321-2.

25. Luong V, Body A, Lal L, Abdulla H, Ahern E, Segelov E. SerOzNET: Assessing COVID-19 vaccine correlates in cancer patients. Asia-Pac J Clin Oncol. 2021;17:202-3.

26. Mohamed S, Lucchini E, Porrazzo M, Ballotta L, De Sabbata GM, Ballerini M, et al. Serological Response to Sars-Cov2 Vaccination in Patients with Hematological Malignancies: Preliminary Data of a Prospective, Multicenter, Observational Study "Cervax". Haematologica. 2021;106(10):123-.

27. Nil A, Olga A, Lucia GP, Merce T, Nerea R, Elisabeth L, et al. Adverse Reactions to Mrna-1273 Sars-Cov-2 Vaccine in Receptor Patients of Allogeneic Hematopoietic Progenitor Transplant. Haematologica. 2021;106(10):322-3.

28. Segelov E. COVID-19 Vaccine response in cancer patients. Asia-Pac J Clin Oncol. 2021;17:107-8.

29. Branagan A, Lei M, Yee AJ, O'Donnell E, Castillo JJ, Raje N, et al. COVID-19 vaccine responsiveness in patients with Multiple Myeloma and Waldenstrom Macroglobulinemia. Clin Lymphoma Myeloma Leuk. 2021;21:S29-S.

30. Malard F, Gaugler B, Gozlan J, Bouquet L, Fofana D, Van De Wyngaert Z, et al. Immunogenicity of SARS-CoV-2 vaccine in patients with multiple myeloma. Clin Lymphoma Myeloma Leuk. 2021;21:S117-S.

31. Shepherd Stc FAALBFWKWMSAMJ-HNSBDRLEKC. 1557O Adaptive immunity to SARS-CoV-2 infection and vaccination in cancer patients: the CAPTURE study. Annals of oncology. 2021;32:S1129-.

32. Shepherd STC, Fendler A, Au L, Byrne F, Wilkinson K, Wu M, et al. Adaptive immunity to SARS-CoV-2 infection and vaccination in cancer patients: The CAPTURE study. Annals of Oncology. 2021;32:S1129-S.

33. Hoornaert E, Dachy F, Hansenne A, Lagneaux E, Gruson D, Bailly S, et al. COVID-19, impact of vaccination in myeloma patients. Clin Lymphoma Myeloma Leuk. 2021;21:S116-S.

34. Ujjani C, Greninger AL, Shadman M, Hill JA, Lynch RC, Warren EH, et al. Heterologous SARS-CoV-2 vaccinations in patients with B-cell lymphoid malignancies. American Journal of Hematology.

35. Saad Albichr I DGJVDNEPXHVVMCBSYJCMSSA. Cellular response to COVID-19 vaccines in hematologic malignancies patients: a new hope for non-responders? Leukemia & lymphoma. 2021:1-4.

36. Harrington P DKJSCSJCFDRSSRKMDADKSORA. Repeated vaccination against SARS-CoV-2 elicits robust polyfunctional T cell response in allogeneic stem cell transplantation recipients. Cancer cell. 2021;39(11):1448-9.

37. Astha T, Jesus Gonzalez L, Niyati G, Radhika G, Lauren CS, Kith P, et al. Seroconversion rates following COVID-19 vaccination amongst patients with malignant disease- the impact of diagnosis and cancer-directed therapies. 2021.

38. University Hospital T. B-pVAC-SARS-CoV-2: Study to Prevent COVID-19 Infection in Adults With Bcell/ Antibody Deficiency. ClinicalTrialsgov. 2021.

39. Thakkar A G-LJDGNGRSLCPKRSKSYKBSRA. Seroconversion rates following COVID-19 vaccination among patients with cancer. Cancer cell. 2021.

40. Thakkar A LJGGNGRSLCPKRSKSYKBSRAK. Seroconversion rates following COVID-19 vaccination amongst patients with malignant disease- the impact of diagnosis and cancer-directed therapies (preprint). Medrxiv. 2021:2021.05.07.21256824.

41. Subbiah Im WLAPASSGBPDADTAGEAFJCRB. Real-world patient-reported and clinical outcomes of BNT162b2 mRNA COVID-19 vaccine in patients with cancer. Journal of clinical oncology. 2021;39(15 SUPPL).

42. Figueiredo JC, Merin N, Ihenacho U, Hamid O, Hendifar AE, Vescio R, et al. Health-related quality of life, vaccine uptake and immune response among cancer patients undergoing treatment during the COVID-19 pandemic. Annals of Oncology. 2021;32:S1150-S.

43. Obermannova R, Demlova R, Selingerova I, Doubek M, Okrouhlicova D, Mlnarikova M, et al. CoVigi phase IV multicentric trial evaluating COVID-19 vaccination adverse events and immune response dynamics in cancer patients: First results on antibody and cellular immunity. Annals of Oncology. 2021;32:S1131-S.

44. Ribera Jm MMCRBPL-LJLMPFMAH-RJMCMG-CI. Characteristics and outcome of adults with acute lymphoblastic leukemia and COVID 19 infection in the first vs. Second epidemic wave in Spain: a study from the Pethema and Geth groups. Hemasphere. 2021;5(SUPPL 2):133-4.

45. Abstracts for the 28th Annual Conference of the German Society for Immunogenetics (Deutsche Gesellschaft fur Immungenetik - DGI). HLA. 2021;98(SUPPL 1).

46. Bonelli M MDTSSDKMMPKBSERHPTHHMMHP. Additional heterologous versus homologous booster vaccination in immunosuppressed patients without SARS-CoV-2 antibody seroconversion after primary mRNA vaccination: a randomized controlled trial. Medrxiv. 2021.

47. Moor M B S-RFHMPADAJMBNLMMCA-SABLR. Humoral and cellular responses to mRNA vaccines against SARS-CoV2 in patients with a history of CD20-B-cell depleting therapy. Medrxiv. 2021:2021.07.04.21259848.

48. Monin L LAGM-RMMDRDMDBIATD-VCHTSGC. Safety and immunogenicity of one versus two doses of the COVID-19 vaccine BNT162b2 for patients with cancer: interim analysis of a prospective observational study. The lancet Oncology. 2021.

49. Moor Mb S-RFHMPADAJMBNLMMCA-SABLR-H. Humoral and cellular responses to mRNA vaccines against SARS-CoV-2 in patients with a history of CD20 B-cell-depleting therapy (RituxiVac): an investigator-initiated, single-centre, open-label study. The lancet Rheumatology. 2021.

50. Mrak D TSKMGMRHSDHPPTHHTRWSBSSK. SARS-CoV-2 vaccination in rituximab-treated patients: b cells promote humoral immune responses in the presence of T-cell-mediated immunity. Annals of the rheumatic diseases. 2021.

51. Nigro O, Sironi G, Bergamaschi L, Gattuso G, Puma N, Livellara V, et al. SARS-CoV-2 vaccination for adolescents and young adult patients treated at a specialist pediatric oncology unit. Pediatr Blood Cancer. 2021;68(10).

52. ZonMw. T2B! immunity after SARS-CoV-2. ICTRP. 2020.

53. Waissengrin B, Agbarya A, Safadi E, Padova H, Wolf I. Short-term safety of the BNT162b2 mRNA COVID-19 vaccine in patients with cancer treated with immune checkpoint inhibitors (vol 22, pg 581, 2021). Lancet Oncol. 2021;22(6):E239-E.

54. Berger T KB. T cells step up after SARS-CoV-2 vaccination with B cell depletion. Nature reviews Neurology. 2021.

55. Chehade L, Zeitoun J, Bejjany R, Charafeddine M, Kreidieh F, Hassan M, et al. COVID-19 vaccination immune response in patients with solid organ and haematologic malignancies: call for active monitoring. ECANCERMEDICALSCIENCE. 2021;15.

56. Da Cruz Tomas Tf EIFVMVRSMADSMCSBSFTMG. 1602P COVID-19 vaccination efficacy in cancer patients: an ongoing prospective trial. Annals of oncology. 2021;32:S1147-.

57. Grinshpun A RYB-DIZDEWDGKL. Serologic response to COVID-19 infection and/or vaccine in cancer patients on active treatment. ESMO open. 2021;6(6):100283.

58. Harrington P, Doores KJ, Saha C, Saunders J, Child F, Dillon R, et al. Repeated vaccination against SARS-CoV-2 elicits robust polyfunctional T cell response in stem cell transplantation recipients. Cancer Cell. 2021;39(11):1448-9.

59. Lane Dl NSSXGWO. COVID-19 Vaccine-Related Axillary and Cervical Lymphadenopathy in Patients with Current or Prior Breast Cancer and Other Malignancies: cross-Sectional Imaging Findings on MRI, CT, and PET-CT. Korean journal of radiology. 2021.

60. Lasagna A, Agustoni F, Percivalle E, Borgetto S, Paulet A, Comolli G, et al. A snapshot of the immunogenity, efficacy and safety of a full course of BNT162b2 anti-SARS-CoV-2 vaccine in cancer patients treated with PD-1/PD-L1 inhibitors: a longitudinal cohort study. ESMO Open. 2021:100272-.

61. Loew B, Tsai R, Hervey J, Hoffman KD, Novack J, Johnson J, et al. Adverse effects of COVID-19 vaccination among cancer patients: Results from an Internet-based survey. Journal of Clinical Oncology. 2021;39(15).

62. Ma Y LNWYZJHYYHWSHZPLJFWWX. Immune checkpoint blocking impact and nomogram prediction of COVID-19 inactivated vaccine seroconversion in patients with cancer: a propensity-score matched analysis. Journal for immunotherapy of cancer. 2021;9(11).

63. Mahase E. Covid-19: pfizer vaccine efficacy was 52% after first dose and 95% after second dose, paper shows. BMJ (Clinical research ed). 2020;371:m4826.

64. Munro C. Covid-19: 40% of patients with weakened immune system mount lower response to vaccines. BMJ (Clinical research ed). 2021;374:n2098.

65. Nelli F FAOAGDSMAGBJRVAMESCCMGP. Effects of active cancer treatment on safety and immunogenicity of COVID-19 mRNA-BNT162b2 vaccine: preliminary results from the prospective observational Vax-On study. Annals of oncology : official journal of the european society for medical oncology. 2021.

66. Passalacqua R, Ratti M, Pan A, Testa S, Molteni A, Tonoli S, et al. Efficacy of SARS-CoV-2 vaccination in cancer patients during treatment: A prospective observational study (ANTICOV trial). Annals of Oncology. 2021;32:S1161-S.

67. Patel M, Felip E, Sharkey R, Krengli M, Chester JD, Sita-Lumsden A, et al. SARS-CoV-2 antibody seroprevalence and safety of vaccines in cancer patients who recovered from COVID-19. Annals of Oncology. 2021;32:S1142-S.

68. Sapir E, Moisa N, Litvin A, Malki E, Vorobiof DA. SARS-CoV-2 vaccines in cancer patients (pts), real-world data (RWD) from 1069 Belong.life users. Annals of Oncology. 2021;32:S1144-S.

69. Thomas SJ, Perez JL, Lockhart SP, Hariharan S, Kitchin N, Bailey R, et al. COVID-19 vaccine in participants (ptcpts) with cancer: Subgroup analysis of efficacy/safety from a global phase III randomized trial of the BNT162b2 (tozinameran) mRNA vaccine. Annals of Oncology. 2021;32:S1129-S.

70. University Health Network T. Immune Response to COVID-19 Vaccine in Immunotherapy (IO) and Non-IO Treated Cancer Patients. ClinicalTrialsgov. 2021.

71. Muñoz FLSACDJJSCJL, Vallejo A, Serrano CSPJCRDIRVH, Martin A, Soria A, Garcia MEORAGLY, et al. Immunogenicity of the mRNA-1273 SARS-CoV-2 vaccine in cancer patients receiving immunotherapy agents. Annals of Oncology. 2021;32:S1424-S.

72. Bacova BKZZIGLKPHTMMNJ. Cellular and Humoral Immune Response to SARS-CoV-2 mRNA Vaccines in Patients Treated with either Ibrutinib or Rituximab (preprint). 2021.

73. Kosiorek PKDHAMRSSS-BA. Systemic COVID-19 vaccination also enhances the humoral immune response after SARS CoV-2 infection in the population of an oncology hospital in Poland. Criteria for COVID-19 re-immunization are needed (preprint). 2021.

74. Di Fusco M, Moran MM, Cane A, Curcio D, Khan F, Malhotra D, et al. Evaluation of COVID-19 vaccine breakthrough infections among immunocompromised patients fully vaccinated with BNT162b2. J Med Econ. 2021;24(1):1248-60.

75. Zoe LL, Myung Sun K, David Xthona L, Hans-Peter R, Vikram R, Janet G, et al. Cellular and humoral Immune response to mRNA COVID-19 vaccination in subjects with chronic lymphocytic leukemia. 2021.

76. Ruggeri EM, Nelli F, Fabbri A, Onorato A, Giannarelli D, Berrios JRGVA, et al. Antineoplastic treatment class modulates COVID-19 mRNA-BNT162b2 vaccine immunogenicity in cancer patients: a secondary analysis of the prospective Vax-On study. ESMO Open. 2021:100350-.

77. Ghandili S, Schonlein M, Lutgehetmann M, Wiesch JSZ, Becher H, Bokemeyer C, et al. Poor post-vaccination anti-SARS-CoV-2-antibody response in patients with Multiple Myeloma correlates with low CD19+B-lymphocyte count and anti-CD38 treatment. Clin Lymphoma Myeloma Leuk. 2021;21:S31-S2.

78. Heudel P, Favier B, Assaad S, Zrounba P, Blay JY. Reduced SARS-CoV-2 infection and death after two doses of COVID-19 vaccines in a series of 1503 cancer patients. Annals of Oncology. 2021;32(11):1443-4.

79. Landre T, Bouharati D, Amara MA, Taleb C. Suboptimal response to COVID-19 mRNA vaccines in older patients with cancer. Annals of Oncology. 2021;32:S1146-S.

80. Linardou H, Spanakis N, Koliou GA, Christopoulou A, Karageorgopoulou S, Alevra N, et al. Responses to SARS-CoV-2 Vaccination in Patients with Cancer (ReCOVer Study): A Prospective Cohort Study of the Hellenic Cooperative Oncology Group. Cancers. 2021;13(18).

81. Loew B TRHJHKDNJJJDDSGSL. Adverse effects of COVID-19 vaccination among cancer patients: results from an Internetbased survey. Journal of clinical oncology. 2021;39(15 SUPPL).

82. Moor MB, Suter-Riniker F, Horn MP, Aeberli D, Amsler J, Moller B, et al. Humoral and cellular responses to mRNA vaccines against SARS-CoV-2 in patients with a history of CD20 B-cell-depleting therapy (RituxiVac): an investigator-initiated, single-centre, open-label study. Lancet Rheumatol. 2021;3(11):E789-E97.

83. Patel M FESRKMCJDS-LAMURBLACJCCA. 1588P SARS-CoV-2 antibody seroprevalence and safety of vaccines in cancer patients who recovered from COVID-19. Annals of oncology. 2021;32:S1142-.

84. Sapir E MNLAMEVDA. 1594P SARS-CoV-2 vaccines in cancer patients (pts), real-world data (RWD) from 1069 Belong.life users. Annals of oncology. 2021;32:S1144-.

85. Thomas Sj PJLLSPHSKNBRLKLETOSUXXD. 1558O COVID-19 vaccine in participants (ptcpts) with cancer: subgroup analysis of efficacy/safety from a global phase III randomized trial of the BNT162b2 (tozinameran) mRNA vaccine. Annals of oncology. 2021;Conference: ESMO Congress 2021. Virtual, Online. 32(Supplement 5):S1129.

86. Wu JTY, La J, Branch-Elliman W, Huhmann LB, Han S, Parmigiani G, et al. Effectiveness of COVID-19 vaccination in cancer patients: A nationwide Veterans Affairs study. Annals of Oncology. 2021;32:S1131-S.

87. Oosting Sf vdVAAMGCHFRSNvBRSDACSEF. mRNA-1273 COVID-19 vaccination in patients receiving chemotherapy, immunotherapy, or chemoimmunotherapy for solid tumours: a prospective, multicentre, non-inferiority trial. The lancet Oncology. 2021.

88. Rosati M TEAMKVBJBRHXPDN-SITIPDMA. Distinct Neutralization Profile of Spike Variants by Antibodies Induced upon SARS-CoV-2 Infection or Vaccination. American journal of hematology. 2021.

89. Tremblay T PJRPSCAMELAFALABR. Response to COVID-19 vaccination in lymphopenic apheresis platelet donors. Transfusion. 2021;61(SUPPL 3):267A-.

90. Lafon AMCCMD-MA-BJP. COVID-19 : adhésion à la vaccination chez des patients immunodéprimés de dermatologie. Annales de Dermatologie et de Vénéréologie - FMC. 2021;1(8, Supplement 1):A332-A3.

91. Gavriatopoulou M, Ntanasis-Stathopoulos I, Korompoki E, Terpos E, Dimopoulos MA. SARS-CoV-2 Vaccines in Patients With Multiple Myeloma. Hemasphere. 2021;5(3).

92. Yap TA, Siu LL, Calvo E, Lolkema MP, LoRusso PM, Soria JC, et al. SARS-CoV-2 vaccination and phase 1 cancer clinical trials. Lancet Oncol. 2021;22(3):298-301.

93. Yekeduz E, Ayasun R, Koksoy EB, Utkan G, Urun Y, Akbulut H. mRNA-based COVID-19 vaccines appear not to increase immune events in cancer patients receiving immune checkpoint inhibitors. Future Virol.

94. Landre T BDAMATC. 1600P Suboptimal response to COVID-19 mRNA vaccines in older patients with cancer. Annals of oncology. 2021;32:S1146-.

95. Lawrence R. Data support third COVID-19 vaccine dose for some patients with cancer. HEM/ONC Today. 2021;22(14):20-.

96. Body AL, Ahern E, Lal L, Luong V, Abdulla H, Segelov E. Seven-day patient-reported toxicity in patients participating in a prospective study of COVID-19 vaccine response in adults with cancer (SerOzNET). Asia-Pac J Clin Oncol. 2021;17:117-.

97. Brest P, Mograbi B, Hofman P, Milano G. COVID-19 vaccination and cancer immunotherapy: should they stick together? Br J Cancer.

98. Terpos E, Rajkumar SV, Leung N. Neutralizing Antibody Testing in Patients With Multiple Myeloma Following COVID-19 Vaccination. JAMA Oncol.

99. COVID-19 Vaccination in Patients with active Cancer. Geburtshilfe Frauenheilkd. 2021;81(04):373-+.

100. Abou-Foul AK, Ross E, Abou-Foul M, George AP. Cervical lymphadenopathy following COVID-19 vaccine: Clinical characteristics and implications for head and neck cancer services. Oral Oncol. 2021;118.

101. Agha Me BMCCWAHG. Suboptimal Response to Coronavirus Disease 2019 Messenger RNA Vaccines in Patients With Hematologic Malignancies: a Need for Vigilance in the Postmasking Era. Open forum infectious diseases. 2021;8(7):ofab353.

102. Benda M MBUHGCSLVARPLTAMHMGKPUFP. Serological SARS-CoV-2 antibody response, potential predictive markers and safety of BNT162b2 mRNA COVID-19 vaccine in haematological and oncological patients. Br J Haematol. 2021.

103. Bird S PASRLTMSRSAWSSKBJKEPCKM. Response to first vaccination against SARS-CoV-2 in patients with multiple myeloma. The Lancet Haematology. 2021.

104. Chebel R, Labaki C, Farhat M, Kattan J. Safety, efficacy and acceptability of SARS-CoV-2 vaccines in patients with cancer. Future Virol. 2021;16(7):443-6.

105. Fong D MMJMM. High levels of anti-SARS-CoV-2 IgG antibodies in previously infected patients with cancer after a single dose of BNT 162b2 vaccine. European journal of cancer (Oxford, England : 1990). 2021;154:4-6.

106. King Da CJGSPSMW. COVID-19 Disease and SARS-CoV-2 Vaccination in Patients with Cancer. Archives of pharmacology and therapeutics. 2021;3(1):5-9.

107. Malard F GBGJBLFDSLEDAOLCRLDRSNvd. Weak immunogenicity of SARS-CoV-2 vaccine in patients with hematologic malignancies. Blood cancer journal. 2021;11(8):142.

108. Mungmungpuntipantip R, Wiwanitkit V. COVID-19 Vaccination in Oncology Patients Receiving Chemotherapy. Clin Oncol. 2021;33(10).

109. Muthukumar R, Kanagavel M, Lu MY, Cristina C, Philip LB, Joseph S-M, et al. Cell-Mediated and Humoral Immune Response to 2-Dose SARS-CoV2 mRNA vaccination in Immunocompromised patient population. 2021.

110. Nierengarten MB. Ongoing Research Examines COVID-19 Vaccination Effectiveness in Patients with Cancer and Survivors. Cancer. 2021;127(18):3281-.

111. Nyberg F FSLMVLHNWBSJSABSGM. Swedish Covid-19 Investigation for Future Insights - A Population Epidemiology Approach Using Register Linkage (SCIFI-PEARL). Clinical epidemiology. 2021;13(pp 649-659):649-59.

112. Ollila Ta LSMRZAPKRRDOAJ. Antibody Response to COVID-19 Vaccination in Adults With Hematologic Malignant Disease. JAMA Oncol. 2021.

113. O'Nions J MLZJR-SCRARCECCPGRKAJCML. SARS-CoV-2 antibody responses in patients with acute leukaemia. Leukemia. 2021;35(1):289-92.

114. Palich R VMMSVAGJMPMAGSJP. Weak immunogenicity after a single dose of SARS-CoV-2 mRNA vaccine in treated cancer patients. Annals of oncology : official journal of the european society for medical oncology. 2021.

115. Re D BJCEDMGLPECAVBPF. Low rate of seroconversion after mRNA anti-SARS-CoV-2 vaccination in patients with hematological malignancies. Leukemia & lymphoma. 2021:1-3.

116. Roeker Le KDATMCNMLSPNDIMSSSCVHBB. COVID-19 vaccine efficacy in patients with chronic lymphocytic leukemia. Leukemia. 2021.

117. Scoccianti S DPCGLBPLAPCSBRFSRSPMPMSM. Acute tolerance of Moderna mRNA-1273 vaccine against COVID-19 in patients with cancer treated with radiotherapy. The lancet Oncology. 2021.

118. So Acp MHTJSKGSMCRBM-IMDSISVHMED. Covid-19 vaccine safety in cancer patients: a single centre experience. Cancers. 2021;13(14).

119. Chen Yw TMDBKEIWTRBIJDB. COVID-19 mRNA vaccines and immune-related adverse events in cancer patients treated with immune checkpoint inhibitors. European journal of cancer (Oxford, England : 1990). 2021;155:291-3.

120. Dhakal B, Abedin S, Fenske T, Chhabra S, Ledeboer N, Hari P, et al. Response to SARS-CoV-2 vaccination in patients after hematopoietic cell transplantation and CAR T-cell therapy. Blood. 2021;138(14):1278-81.

121. Gambichler T, Boms S, Hessam S, Tischoff I, Tannapfel A, Luttringhaus T, et al. Primary cutaneous anaplastic large-cell lymphoma with marked spontaneous regression of organ manifestation after SARS-CoV-2 vaccination. Br J Dermatol.

122. Malissen N, Ninove L, de Lamballerie X, Andre N, Gaudy-Marqueste C. Safety and immunogenicity after 2 doses of the BNT162b2 COVID-19 vaccine in an early-phase oncology trial centre population. Eur J Cancer. 2021;156:125-6.

123. Panou E, Nikolaou V, Marinos L, Kallambou S, Sidiropoulou P, Gerochristou M, et al. Recurrence of cutaneous T-cell lymphoma post viral vector COVID-19 vaccination. J Eur Acad Dermatol Venereol.

124. Schoemans H, Ljungman P. One step at a time. Blood. 2021;138(14):1208-9.

125. Slomski A. Most Fully Vaccinated Patients With Cancer Have SARS-CoV-2 Antibodies. JAMA-J Am Med Assoc. 2021;326(9):800-.

126. Sun C, Pleyer C, Wiestner A. COVID-19 vaccines for patients with haematological conditions. Lancet Haematol. 2021;8(5):E312-E4.

127. Wu JTY, La J, Branch-Elliman W, Huhmann LB, Han SS, Parmigiani G, et al. Association of COVID-19 Vaccination With SARS-CoV-2 Infection in Patients With Cancer A US Nationwide Veterans Affairs Study. JAMA Oncol.

128. Zemel M, Kian W, Kestenbaum EH, Alguayn W, Levitas D, Sharb AA, et al. Safety of the BNT162b2 mRNA COVID-19 vaccine in oncologic patients undergoing numerous cancer treatment options. Annals of Oncology. 2021;32:S1142-S3.

129. Easdale S, Shea R, Ellis L, Bazin J, Davis K, Dallas F, et al. Serologic Responses following a Single Dose of SARS-Cov-2 Vaccination in Allogeneic Stem Cell Transplantation Recipients. Transpl Cell Ther. 2021;27(10).

130. Sengsayadeth S, Broglie L, Savani BN. Effectiveness of COVID-19 vaccination in patients after allogeneic haematopoietic cell transplant: how much protection are we getting? Br J Haematol.

131. Chung DJ, Shah GL, Devlin SM, Ramanathan LV, Doddi S, Pessin MS, et al. Disease- and Therapy-Specific Impact on Humoral Immune Responses to COVID-19 Vaccination in Hematologic Malignancies. Blood Cancer Discov. 2021;2(6):568-76.

132. Embi Pj LMENALPPGMNKDKOTCKNPLIC. Effectiveness of 2-Dose Vaccination with mRNA COVID-19 Vaccines Against COVID-19-Associated Hospitalizations Among Immunocompromised Adults - Nine States, January-September 2021. MMWR Morbidity and mortality weekly report. 2021;70(44):1553-9.

133. Amsterdam UMClV. COBRA-KAI study: COVID-19 vaccination in patients with a hematological disease. ICTRP. 2021.

134. Assistance Publique - Hopitaux de P. Study of the Humoral Response to SARS-CoV-2 Variants and of the Cellular Response After Vaccination Against COVID-19 in Immunocompromised People. ClinicalTrialsgov. 2021.

135. Assistance Publique - Hopitaux de P. Anti-Sars-Cov2 Vaccine Efficacy in Patients With Malignant Pathologies. ClinicalTrialsgov. 2021.

136. Auxilio Mutuo Cancer C. Antibodies Production After Covid-19 Vaccination Among Patients With Medical History of Cancer and Anti-CD-20 Treatment. ClinicalTrialsgov. 2021.

137. Azienda Socio Sanitaria Territoriale di C. Effectiveness of mRNA Covid-19 Vaccines on Cancer Patients:Observational Study. (ANTICOV). ClinicalTrialsgov. 2021.

138. Barbara Ann Karmanos Cancer I. COVID-19 VAX Booster Dosing in Patients With Hematologic Malignancies. ClinicalTrialsgov. 2021.

139. Bezmialem Vakif U. Evaluation of the Effect and Side Effect Profile of Covid-19 Vaccine in Cancer Patients. ClinicalTrialsgov. 2021.

140. Centre Henri B. Efficacy of COVID-19 Vaccination in Patientstreated With Anti-CD20 for Follicular Lymphoma or Mantle Cell Lymphoma. ClinicalTrialsgov. 2021.

141. Centre Hospitalier de C. Efficacy and Safety of the Anti- COVID-19 Vaccin in Clinical Hematology Patients. ClinicalTrialsgov. 2021.

142. Charite - Universitatsmedizin B. Immune response of COVID-19 vaccines in medical staff and special risk populations. ICTRP. 2021.

143. City of Hope Medical C. SARS-CoV-2 Vaccine (COH04S1) Versus Emergency Use Authorization SARS-COV-2 Vaccine for the Treatment of COVID-19 in Patients With Blood Cancer. ClinicalTrialsgov. 2021.

144. Hellenic Cooperative Oncology G. Responses to COVID-19 Vaccination in Patients With Cancer. ClinicalTrialsgov. 2021.

145. Ente Ospedaliero Ospedali G. BNT162b2 Messenger Ribonucleic Acid (mRNA) Covid-19 Vaccine in Cancer Patients on Active Treatment. ClinicalTrialsgov. 2021.

146. Fukuoka B, Marrow Transplantation G. Observational study on efficacy and safety of vaccines against the novel coronavirus disease (COVID-19) in hematopoietic stem cell transplantation (HSCT) patient. ICTRP. 2021.

147. Henry Ford Health S. Response to SARS-CoV-2 Vaccine in Stem Cell Transplant and Cellular Therapy Patients. ClinicalTrialsgov. 2021.

148. Ilaria C. A Study on the Immune-response to COVID-19 Vaccination in Cancer Patients - the IOSI-COVID-19-001 Study. ClinicalTrialsgov. 2021.

149. Indiana U. Immune Responses in Oncology Patients to Novel Coronavirus Vaccines (IROC). ClinicalTrialsgov. 2021.

150. Larry Saltzman MD. The Leukemia and Lymphoma Society (LLS) T-cells in Blood Cancer and COVID-19. ClinicalTrialsgov. 2021.

151. Liege CHUd. Impact of the immune system on response to COVID-19 vaccine in allogeneic stem cell recipients. ICTRP. 2021.

152. Massachusetts General H. Vaccine Responsiveness in Patients With Chronic Lymphocytic Leukemia. ClinicalTrialsgov. 2021.

153. Massachusetts General H. Covid-19 Vaccine Responsiveness in MM and Waldenstrom. ClinicalTrialsgov. 2021.

154. Mayo C. Immunogenicity and Safety of Commercially Available Vaccines Against SARS-CoV-2 (COVID-19) in Patients With Hematologic Malignancies. ClinicalTrialsgov. 2021.

155. Medical University of G. Humoral (antibodies) and cellular (white blood cells) immune response to COVID-19 vaccines in immunocompromised (people with impaired immune system) and healthy individuals – The CoVVac study. ICTRP. 2021.

156. Medical University of V. A Randomized, Parallel Group, Single-Blind, Phase 2 Study to Evaluate the immune response of two classes of SARS-Cov-2 Vaccines employed as Second Boost in Patients under current Rituximab Therapy and no humoral response after standard mRNA vaccination. ICTRP. 2021.

157. Monika J. Coronavirus Disease 2019 (COVID-19) Vaccination in Cancer Patients. ClinicalTrialsgov. 2021.

158. Montefiore Medical C. Booster Dose Trial. ClinicalTrialsgov. 2021.

159. Mumc. SARS-CoV-2 vaccination response in patients with haematological disease. ICTRP. 2021.

160. National Cancer I. A Trial of the Safety and Immunogenicity of the COVID-19 Vaccine (mRNA-1273) in Participants With Hematologic Malignancies and Various Regimens of Immunosuppression, and in Participants With Solid Tumors on PD1/PDL1 Inhibitor Therapy. ClinicalTrialsgov. 2021.

161. Oslo University H. Booster dose of SARS-CoV-2 vaccine to patients with suboptimal response to previous doses. ICTRP. 2021.

162. Pontificia Universidad Catolica de C. Immune Response to Anti-SARSCoV2 Vaccine in Immunocompromised Patients: a Cohort Study. ClinicalTrialsgov. 2021.

163. Royal B, Women's H. COVID-19 Vaccine Efficacy in patients with Blood Cancer. ICTRP. 2021.

164. Seoul National University H. Immunogenicity and Safety of COVID-19 Vaccine in Cancer Patients. ClinicalTrialsgov. 2021.

165. Shonan Fujisawa Tokushukai H. Immunogenicity and safety of severe acute respiratory syndrome coronavirus 2 vaccination in lung cancer patients receiving immune checkpoint inhibitors: a multicenter observational study in Japan. ICTRP. 2021.

166. Stadtisches Klinikum Dessau. Medizinische Hochschule Brandenburg Theodor F. Monitoring the immune response of anti-SARS-CoV2 vaccinated individuals and COVID-19-positive patients – a prospective monocentric non-interventional observational study. ICTRP. 2021.

167. The University of Texas Health Science Center at San A. Host Immune Response to Novel RNA COVID Vaccination. ClinicalTrialsgov. 2021.

168. Tokyo Metropolitan C, Infectious Diseases Center Komagome H. A prospective observational study to investigate the safety of SARS-Cov-2 vaccine for patients with advanced lung cancer who are treated with immune checkpoint inhibitor. ICTRP. 2021.

169. Universitatsklinikum H-E. Immunity Against SARS-CoV-2 Corona Virus in the Oncology Outpatient Setting. ClinicalTrialsgov. 2021.

170. University Hospital A. The Immune Reaction Upon COVID-19 Vaccination in the Belgian Cancer Population. ClinicalTrialsgov. 2021.

171. University Hospitals of North Midlands NHST. Covid-19 Vaccine Response in Immunocompromised Haematology Patients. ClinicalTrialsgov. 2021.

172. University Medical Center G. Vaccination against cOvid In CancEr. ICTRP. 2021.

173. University Medical Center G. Vaccination Against COVID-19 in Cancer. ClinicalTrialsgov. 2021.

174. University of A. Immune Response to the COVID-19 Vaccine. ClinicalTrialsgov. 2021.

175. University of B. Observational cohort trial of immune response in patients with chronic health conditions following coronavirus vaccination. ISRCTN. 2021.

176. University of Kansas Medical C. To Assess Immunogenicity of Coronavirus Disease 2019 (COVID-19) Vaccine in Cancer Patients Receiving Cancer Treatment. ClinicalTrialsgov. 2021.

177. University of L. Impact of the Immune System on Response to Anti-Coronavirus Disease 19 (COVID-19) Vaccine in Allogeneic Stem Cell Recipients (Covid Vaccin Allo). ClinicalTrialsgov. 2021.

178. University of R. Serologic Response to the SARS-CoV-2 mRNA-1273 Vaccine in Select Subsets of Oncology Patients. ClinicalTrialsgov. 2021.

179. Medizin I UF. Humoral and Cellular Immune Response to Commercially Available Vaccines Against SARS-CoV-2 (COVID-19) in Patients with Hematologic and Solid Malignancies. ICTRP. 2021.

180. Efficace F, Breccia M, Fazi P, Cottone F, Holzner B, Vignetti M. The GIMEMA-ALLIANCE Digital Health Platform for Patients With Hematologic Malignancies in the COVID-19 Pandemic and Postpandemic Era: Protocol for a Multicenter, Prospective, Observational Study. JMIR RES Protoc. 2021;10(6).

181. Istituti Fisioterapici O. Immune response to the BNT162b2 vaccine against the COVID-19 virus in health-care-workers and immunocompromised patients. ICTRP. 2021.

182. Karolinska U. Immunologiskt svar efter vaccinering med mRNA vaccin mot covid-19, Comirnaty, hos immunsupprimerade och immunkompetenta personer. En oppen icke randomiserad multicenterstudie i fas IV. ICTRP. 2021.

183. Bio NSE. Study to Evaluate Safety, Tolerability & Immunogenicity of BNT162b2 in Immunocompromised Participants ≥2 Years. ClinicalTrialsgov. 2021.

184. Blood Disorders Center AH. Evaluation of clinical efficacy of COVID-19 vaccination in patients with hematological diseases. ICTRP. 2021.

185. Center HLMC, Research I. Immunogenicity of a Third Dose of mRNA-1273 Vaccine Among Cancer Patients. ClinicalTrialsgov. 2021.

186. Hamamatsu University School of M. Analysis of immune response behavior and agents by vaccination with new coronavirus infections in patients with hematopoietic malignancies. ICTRP. 2021.

187. Jules Bordet I. Immune Response in Patients With Cancer Undergoing mRNA Vaccination Against SARS-CoV-2. ClinicalTrialsgov. 2021.

188. Monash H. The SerOzNET study seeks to better understand the safety and efficacy of the COVID-19 vaccines in people with cancer. ICTRP. 2021.

189. Pontificia Universidad Catolica de C. Immune Response to Third Dose of SARS-CoV-2 Vaccine in a Cohort of Cancer Patients on Active Treatment. ClinicalTrialsgov. 2021.

190. University of B. Randomised trial optimising COVID-19 vaccination in patients with chronic health conditions and a poor response to standard vaccination. ICTRP. 2021.

191. University of Milano B. Evaluation of the Immune Response to Sars-Cov-2 Vaccines in Haematological Patients: Prospective Single Center Study. ClinicalTrialsgov. 2021.

192. Ram R, Hagin D, Kikozashvilli N, Freund T, Amit O, Bar-On Y, et al. Safety and Immunogenicity of the BNT162b2 mRNA COVID-19 Vaccine in Patients after Allogeneic HCT or CD19-based CART therapy-A Single-Center Prospective Cohort Study. Transpl Cell Ther. 2021;27(9):788-94.

193. Tel-Aviv Sourasky Medical C. COVID-19 Vaccine in Patients After Allogeneic HCT, CAR-T Therapy and With Primary Immune Deficiency. ClinicalTrialsgov. 2021.

194. Karthik R, Ross S, Sally J, Paul W, Sherin V, Alison T, et al. Immune response to COVID-19 vaccination is attenuated by poor disease control and antimyeloma therapy with vaccine driven divergent T cell response. 2021.

195. Karthik R, Ross S, Sally J, Sherin V, Alison T, Jemma L, et al. COVID symptoms, testing, shielding impact on patient reported outcomes and early vaccine responses in individuals with multiple myeloma. 2021.

196. Crombie JL, Sherman AC, Cheng CA, Ryan CE, Zon R, Desjardins M, et al. Activity of mRNA COVID-19 vaccines in patients with lymphoid malignancies COMMENT. Blood Adv. 2021;5(16):3062-5.

197. Addeo A SPKBNHRDABDMMKVDPYTBSSP. Immunogenicity of SARS-CoV-2 messenger RNA vaccines in patients with cancer. Cancer cell. 2021.

198. Avivi I BRSTSGMMAAL-SNTSPCBNMMTYB. Humoral response rate and predictors of response to BNT162b2 mRNA COVID19 vaccine in patients with multiple myeloma. Br J Haematol. 2021.

199. Herishanu Y AIAASGLSBYMMMZ-BTSYSLJEPC. Efficacy of the BNT162b2 mRNA COVID-19 Vaccine in Patients with Chronic Lymphocytic Leukemia. Blood. 2021.

200. Herishanu Y AILSSGBYMMMZ-BTSLPCGP. Six Months Antibody Persistence after BNT162b2 mRNA COVID-19 Vaccination in Patients with Chronic Lymphocytic Leukemia. Blood Adv. 2021.

201. Tel-Aviv Sourasky Medical C. The Capability of Haemato-oncology Patients to Generate Antibodies Against COVID-19. ClinicalTrialsgov. 2021.

202. Soledad H, Jeremie Z, Timothee B, Amani O, Delphine P, Paul D, et al. Anti-CD38 therapy impairs SARS-CoV-2 vaccine response in multiple myeloma patients. 2021.

203. Ghady H, Mounzer A, Amy L, Kelsey L, Rachel T, Andrew B, et al. Immunogenicity of COVID-19 Vaccination in Immunocompromised Patients: An Observational, Prospective Cohort Study Interim Analysis. 2021.

204. Del Poeta G, Bomben R, Polesel J, Rossi FM, Pozzo F, Zaina E, et al. COVID-19 vaccination: Evaluation of risk for protection failure in chronic lymphocytic leukemia patients. Hematol Oncol.

205. Ghione P, Gu JJ, Attwood K, Torka P, Goel S, Sundaram S, et al. Impaired humoral responses to COVID-19 vaccination in patients with lymphoma receiving B-cell-directed therapies. Blood. 2021;138(9):811-4.

206. Stampfer Sd GMSJSBSRBDDCHXNLMGTFEAE. Response to mRNA vaccination for COVID-19 among patients with multiple myeloma. Leukemia. 2021.

207. Ehmsen S AAJSSNACØSVHJUSJISFHDHJ. Antibody and T cell immune responses following mRNA COVID-19 vaccination in patients with cancer. Cancer cell. 2021;39(8):1034-6.

208. Eifer M TNAYKNDLSJKNGMEY. Covid-19 mRNA Vaccination: age and Immune Status and its Association with Axillary Lymph Node PET/CT Uptake. Journal of nuclear medicine. 2021.

209. Gavriatopoulou M, Terpos E, Malandrakis P, Ntanasis-Stathopoulos I, Briasoulis A, Gumeni S, et al. Myeloma patients with COVID-19 have superior antibody responses compared to patients fully vaccinated with the BNT162b2 vaccine. Br J Haematol.

210. Gavriatopoulou M TEMPN-SIBAGSFDPEDMMTFE-PEK. Myeloma patients with COVID-19 have superior antibody responses compared to patients fully vaccinated with the BNT162b2 vaccine. Br J Haematol. 2021.

211. Gavriatopoulou M TEN-SIBAGSMPFDMMTFE-PEKNKE. Poor neutralizing antibody responses in 106 patients with WM after vaccination against SARS-CoV-2; a prospective study. Blood Adv. 2021.

212. Terpos E, Gavriatopoulou M, Fotiou D, Giatra C, Asimakopoulos I, Dimou M, et al. Poor Neutralizing Antibody Responses in 132 Patients with CLL, NHL and HL after Vaccination against SARS-CoV-2: A Prospective Study. CANCERS. 2021;13(17).

213. Terpos E, Gavriatopoulou M, Ntanasis-Stathopoulos I, Briasoulis A, Gumeni S, Malandrakis P, et al. Patients with Multiple Myeloma on treatment with Anti-CD38 or Anti-BCMA agents have a suboptimal humoral response following COVID-19 vaccination. Clin Lymphoma Myeloma Leuk. 2021;21:S104-S.

214. Terpos E GMN-SIBAGSMPFDPEDMMTFRME-P. The neutralizing antibody response post COVID-19 vaccination in patients with myeloma is highly dependent on the type of anti-myeloma treatment. Blood cancer journal. 2021;11(8):138.

215. Terpos E KVN-SIGMGSMPPEDKETIPDMA. Robust Neutralizing Antibody Responses 6 Months Post Vaccination with BNT162b2: a Prospective Study in 308 Healthy Individuals. Life (Basel, Switzerland). 2021;11(10).

216. Terpos E PMN-SIKVMEFDGMMPKETIPDMA. High prevalence of anti-pf4 antibodies following chadox1 ncov-19 (Azd1222) vaccination even in the absence of thrombotic events. Vaccines. 2021;9(7).

217. Terpos E TIPPICISADAFN-SIPEDGSLCI. Development of neutralizing antibodies against SARS-COV-2 in healthy sanitary workers and octogenarians post-BNT162B2 MRNA covid-19 vaccine: first results of a prospective trial in 366 individuals. Hemasphere. 2021;5(SUPPL 2):98-.

218. Terpos EGMFDGCAIDMSAD, Ntanasis-Stathopoulos IDIBAKEAMBIPPTIP, Vassilakopoulos TP, Pagoni MDMA. Poor Neutralizing Antibody Responses in 132 Patients with CLL, NHL and HL after Vaccination against SARS-CoV-2: A Prospective Study. Cancers. 2021;13(17):4480-.

219. National, Kapodistrian University of A. Study of the Kinetics of Antibodies Against COVID-19 (SARS-CoV-2) and of Cellular Subpopulations of the Immune System. ClinicalTrialsgov. 2021.

220. Benjamini O RLIGBASLGNSSDNAARGLYBDSS. Safety and efficacy of BNT162b mRNA Covid19 Vaccine in patients with chronic lymphocytic leukemia. Haematologica. 2021.

221. Bnai Zion Medical C. Safety, Efficacy of BNT162b2 mRNA Vaccine in CLL. ClinicalTrialsgov. 2021.

222. Maneikis K SKRUVVCRKLNDBVPVBTGL. Immunogenicity of the BNT162b2 COVID-19 mRNA vaccine and early clinical outcomes in patients with haematological malignancies in Lithuania: a national prospective cohort study. The Lancet Haematology. 2021.

223. Vilnius U. Analysis of Immunogenicity, Safety and Efficacy of COVID-19 Vaccines in Immunosuppressed Individuals. ClinicalTrialsgov. 2021.

224. Jurgens Em KTJZZJSMSCBSAFEKPJGANEB. Serologic response to mRNA COVID-19 vaccination in lymphoma patients. American journal of hematology. 2021.

225. Cohen D HKSCYCPCAIHYE-SE. Correlation between BNT162b2 mRNA Covid-19 vaccine-associated hypermetabolic lymphadenopathy and humoral immunity in patients with hematologic malignancy. European journal of nuclear medicine and molecular imaging. 2021.

226. Cohen D HKSWIE-SE. A sigh of relief: vaccine-associated hypermetabolic lymphadenopathy following the third COVID-19 vaccine dose is short in duration and uncommonly interferes with the interpretation of [18F]FDG PET-CT studies performed in oncologic patients. European journal of nuclear medicine and molecular imaging. 2021.

227. Lim Sh CNJMJ-PDCGPOCAFCPAMJPWMG. Antibody responses after SARS-CoV-2 vaccination in patients with lymphoma. The Lancet Haematology. 2021.

228. Lim Sh CNJ-PDJMMCCHWTMBLRBAWNMDF. Serological responses after SARS-CoV-2 vaccination first dose in patients with lymphoid malignancy: first interim analysis of the UK proseco study. Hematol Oncol. 2021;39(SUPPL 2):381-2.

229. Sean Hua L, Nicola C, Marina J, Debora J-P, Graham PC, Ann OC, et al. Antibody Responses after SARS-CoV-2 Vaccination in Lymphoma. 2021.

230. University Hospital Southampton NHSFT. Immune Responses to COVID-19 Vaccination in Lymphoma Patients. ClinicalTrialsgov. 2021.

231. Madhumita S, Ellen F, Cyril G, Vincent N, Sarah B, Isobel B, et al. Spike-antibody responses following first and second doses of ChAdOx1 and BNT162b2 vaccines by age, gender, and clinical factors - a prospective community cohort study (Virus Watch). 2021.

232. Greenberger LM, Saltzman LA, Senefeld JW, Johnson PW, DeGennaro LJ, Nichols GL. Anti-spike antibody response to SARS-CoV-2 booster vaccination in patients with B cell-derived hematologic malignancies. Cancer Cell. 2021;39(10):1297-9.

233. Greenberger Lm SLASJWJPWDLJNGL. Antibody response to SARS-CoV-2 vaccines in patients with hematologic malignancies. Cancer cell. 2021;39(8):1031-3.

234. Lymphoma, Leukemia S. The Lymphoma and Leukemia Society COVID-19 Registry. ClinicalTrialsgov. 2021.

235. Gurion R RUIGG-GALCRPB-ZHSMT-AMDEJHN. Humoral serologic response to the BNT162b2 vaccine is abrogated in lymphoma patients within the first 12 months following treatment with anti-CD2O antibodies. Haematologica. 2021.

236. Marchesi F, Pimpinelli F, Sperandio E, Papa E, Falcucci P, Pontone M, et al. The 12-week kinetics of anti-SARS-CoV-2 antibodies in different haematological cancers after vaccination with BNT162b2. Br J Haematol.

237. Pimpinelli F, Marchesi F, Piaggio G, Giannarelli D, Papa E, Falcucci P, et al. Lower response to BNT162b2 vaccine in patients with myelofibrosis compared to polycythemia vera and essential thrombocythemia. J Hematol Oncol. 2021;14(1).

238. Pimpinelli F MFPGGDPEFPPMDMSLVLMADDEG. Fifth-week immunogenicity and safety of anti-SARS-CoV-2 BNT162b2 vaccine in patients with multiple myeloma and myeloproliferative malignancies on active treatment: preliminary data from a single institution. J Hematol Oncol. 2021;14(1):81.

239. Marchesi F PFGDRLPEFPPMDDEGdMSLVMCC. Impact of anti-CD20 monoclonal antibodies on serologic response to BNT162b2 vaccine in B-cell Non-Hodgkin’s lymphomas. Leukemia. 2021.

240. Mona A, Pejman P, Babak P, Danial F-P, Mansoureh D, Maryam N, et al. Immunogenicity and Safety of the inactivated SARS-CoV-2 vaccine (BBIBP-CoV) in patients with malignancy. 2021.

241. Perry C LEBRSGMMMAATYCYCBNB-KONM. Efficacy of the BNT162b2 mRNA COVID-19 vaccine in patients with B-cell non-Hodgkin lymphoma. Blood Adv. 2021;5(16):3053-61.

242. Peter B, Ola B, Lotta H, Stephan M, Piotr N, Puran C, et al. Safety and efficacy of the mRNA BNT162b2 vaccine against SARS-CoV-2 in five groups of immunocompromised patients and healthy controls in a prospective open-label clinical trial. 2021.

243. Karolinska University H. COVID-19 Vaccination of Immunodeficient Persons (COVAXID). ClinicalTrialsgov. 2021.

244. Tadmor T BOBARGRL. Antibody persistence 100 days following the second dose of BNT162b mRNA Covid19 vaccine in patients with chronic lymphocytic leukemia. Leukemia. 2021.

245. Fox Ta KAAELORMASDSSONJKJVETWBS. Low seropositivity and suboptimal neutralisation rates in patients fully vaccinated against COVID-19 with B-cell malignancies. Br J Haematol. 2021.

246. Tzarfati Kh GOAAR-LNSMHLB-LPCABK-MM. BNT162b2 COVID-19 Vaccine is significantly less effective in patients with hematologic malignancies. American journal of hematology. 2021.

247. Aleman A UBTKKKGCRBKASSKPVISSGVOO. Variable cellular responses to SARS-CoV-2 in fully vaccinated patients with multiple myeloma. Cancer cell. 2021;39(11):1442-4.

248. Attolico I TFCPSCPDMGVPPTGFLCGASNC. Serological response following BNT162b2 anti-SARS-CoV-2 mRNA vaccination in haematopoietic stem cell transplantation patients. Br J Haematol. 2021.

249. Bitoun S HJV-FCDNBRMLJCDDBMLGRSRRA. Response to COVID-19 mRNA vaccination in multiple myeloma is conserved but impaired compared to controls. J Hematol Oncol. 2021;14(1):166.

250. Mairhofer M KLKSGRSMKKPMRISHJFDSHAP. Humoral and cellular immune responses in SARS-CoV-2 mRNA-vaccinated patients with cancer. Cancer cell. 2021.

251. Lockmer S UKKMSCMKF-TEAELJNH. Antibody response to COVID-19 mRNA vaccine (Comirnaty) in myeloma patients treated with high-dose melphalan and/or immunotherapy. American journal of hematology. 2021.

252. Canti L H-BSDINJPPHLHASSWEEGGSSLM. Predictors of neutralizing antibody response to BNT162b2 vaccination in allogeneic hematopoietic stem cell transplant recipients. J Hematol Oncol. 2021;14(1):174.

253. Marasco V CCGAFLMMMRCLVPLSSFMDAGIG. T-cell immune response after mRNA SARS-CoV-2 vaccines is frequently detected also in the absence of seroconversion in patients with lymphoid malignancies. Br J Haematol. 2021.

254. Cattaneo D, Bucelli C, Cavallaro F, Consonni D, Iurlo A. Impact of diagnosis and treatment on response to COVID-19 vaccine in patients with BCR-ABL1-negative myeloproliferative neoplasms. A single-center experience. Blood Cancer Journal. 2021;11(11).

255. McKenzie Dr M-RMMLATLTA-JSGCPEGRSKSJTC. Humoral and cellular immunity to delayed second dose of SARS-CoV-2 BNT162b2 mRNA vaccination in patients with cancer. Cancer cell. 2021;39(11):1445-7.

256. Chan Wy HLWWSEALCSJDECNLCSYMAWB. Serological response to the BNT162b2 mRNA or ChAdOx1 nCoV-19 COVID-19 vaccine after first and second doses in patients with plasma cell disorders: influence of host and disease factors. Br J Haematol. 2021.

257. Chiarucci M PSIAGBLFCMVG. Immunological Response Against SARS-COV-2 After BNT162b2 Vaccine Administration Is Impaired in Allogeneic but Not in Autologous Stem Cell Transplant Recipients. Frontiers in oncology. 2021;11:737300.

258. Molica S GDLMZDMALDGVPGGIMPTFLL. Efficacy of the BNT162b2 mRNA COVID-19 Vaccine in Patients with Chronic Lymphocytic Leukemia: a Serologic and Cellular Study. Chemotherapy. 2021.

259. Peeters M VLTLVGVKSPBRSVdMIDKSDYHM. Reduced humoral immune response after BNT162b2 coronavirus disease 2019 messenger RNA vaccination in cancer patients under antineoplastic treatment. ESMO open. 2021;6(5):100274.

260. Fendler A, Au L, Shepherd STC, Byrne F, Cerrone M, Boos LA, et al. Functional antibody and T cell immunity following SARS-CoV-2 infection, including by variants of concern, in patients with cancer: the CAPTURE study. Nat Cancer.

261. Fendler A, Shepherd STC, Au L, Wilkinson KA, Wu M, Byrne F, et al. Adaptive immunity and neutralizing antibodies against SARS-CoV-2 variants of concern following vaccination in patients with cancer: the CAPTURE study. Nat Cancer.

262. Fendler A ALBLABFSSTCSBGCLWBXWCMC. Adaptive immunity to SARS-CoV-2 in cancer patients: the CAPTURE study (preprint). Medrxiv. 2020:2020.12.21.20248608.

263. Zeng C EJPRSWJLCBZERMPCKSLOEMG. Impaired Neutralizing Antibody Response to COVID-19 mRNA Vaccines in Cancer Patients. Medrxiv. 2021.

264. Zeng C EJPRSWJLCBZERMPCKSLOEMG. Impaired neutralizing antibody response to COVID-19 mRNA vaccines in cancer patients. Cell & bioscience. 2021;11(1):197.

265. Pinana Jl L-CLMRMJVLPAM-MGF-MAFEPMJS-L, Cell Therapy G. SARS-CoV-2-reactive antibody detection after SARS-CoV-2 vaccination in hematopoietic stem cell transplant recipients: prospective survey from the Spanish Hematopoietic Stem Cell Transplantation and Cell Therapy Group. American journal of hematology. 2021.

266. Yeshurun M POSLYDB-ZHRMS-NMWORPRU. Humoral serologic response to the BNT162b2 vaccine after allogeneic haematopoietic cell transplantation. Clinical microbiology and infection. 2021.

267. Yanodng S, Jane AF, Juliette H, Ann S, Kartik N, Asha S, et al. COVID-19 Vaccine Failure in Chronic Lymphocytic Leukemia and Monoclonal B-Lymphocytosis; Humoral and Cellular Immunity. 2021.

268. Rahav G LYLJOBMHHTNS-TSESDMB-AZHRIV. BNT162b2 mRNA COVID-19 vaccination in immunocompromised patients: a prospective cohort study. EClinicalMedicine. 2021;41:101158.

269. Figueiredo Jc MNMHOCSYLTCWNNFLJFJDJGJ. Longitudinal SARS-CoV-2 mRNA vaccine-induced humoral immune responses in cancer patients. Cancer research. 2021.

270. Redjoul R LBABFFSMS. Antibody response after second BNT162b2 dose in allogeneic HSCT recipients. Lancet. 2021;398(10297):298-9.

271. Tamari R, Politikos I, Knorr DA, Vardhana SA, Young JC, Marcello LT, et al. Predictors of Humoral Response to SARS-CoV-2 Vaccination after Hematopoietic Cell Transplantation and CAR T-cell Therapy. Blood Cancer Discov. 2021;2(6):577-85.

272. Reimann P UHMBBMSLVALTAMHMGKGCMSFP. Efficacy and safety of heterologous booster vaccination with Ad26.COV2.S after BNT162b2 mRNA COVID-19 vaccine in haemato-oncological patients with no antibody response. Br J Haematol. 2021.

273. Sherman Ac DMCCABBINZGKJTNWDRSRHVT. SARS-CoV-2 mRNA Vaccines in Allogeneic Hematopoietic Stem Cell Transplant Recipients: immunogenicity and Reactogenicity. Clinical infectious diseases. 2021.

274. Kozak Ke OLDASAMSJFCCJDRJMSMMJRR. Serum antibody response in patients with philadelphia-chromosome positive or negative myeloproliferative neoplasms following vaccination with SARS-CoV-2 spike protein messenger RNA (mRNA) vaccines. Leukemia. 2021.

275. Lindemann M KVTLFNT-MNDMSSKHRHCHPAKM. Humoral and cellular vaccination responses against sars-cov-2 in hematopoietic stem cell transplant recipients. Vaccines. 2021;9(10).

276. Shem-Tov N YRDILVLIOLLYAANASARG. Immunogenicity and safety of the BNT162b2 mRNA COVID-19 vaccine in haematopoietic stem cell transplantation recipients. Br J Haematol. 2021.

277. Schiller Salton N SMTIHNAZTHNS-MYHKLN. Attenuated humoral immune response following anti-SARS-CoV-2 vaccine in heavily pretreated patients with multiple myeloma and AL amyloidosis. American journal of hematology. 2021.

278. Salvini M MFDCMLBAMBBMMRIASDBBASGM. Immunogenicity of anti-SARS-CoV-2 Comirnaty vaccine in patients with lymphomas and myeloma who underwent autologous stem cell transplantation. Bone marrow transplantation. 2021.

279. Ospedale di Circolo - Fondazione M. Immunogenicity of Covid-19 Vaccination for Patients With Hematological Malignancies. ClinicalTrialsgov. 2021.
