# Supplementary material for: Effectiveness, immunogenicity, and safety of COVID-19 vaccines for individuals with hematological malignancies: a systematic review
Source: Blood Cancer J. 2022 May 31;12(5):86. doi: 10.1038/s41408-022-00684-8 (PMC9152308; doi:10.1038/s41408-022-00684-8)
Supplement: Supplementary file 4 — supplementary figure 1 [file 41408_2022_684_MOESM4_ESM.pdf]

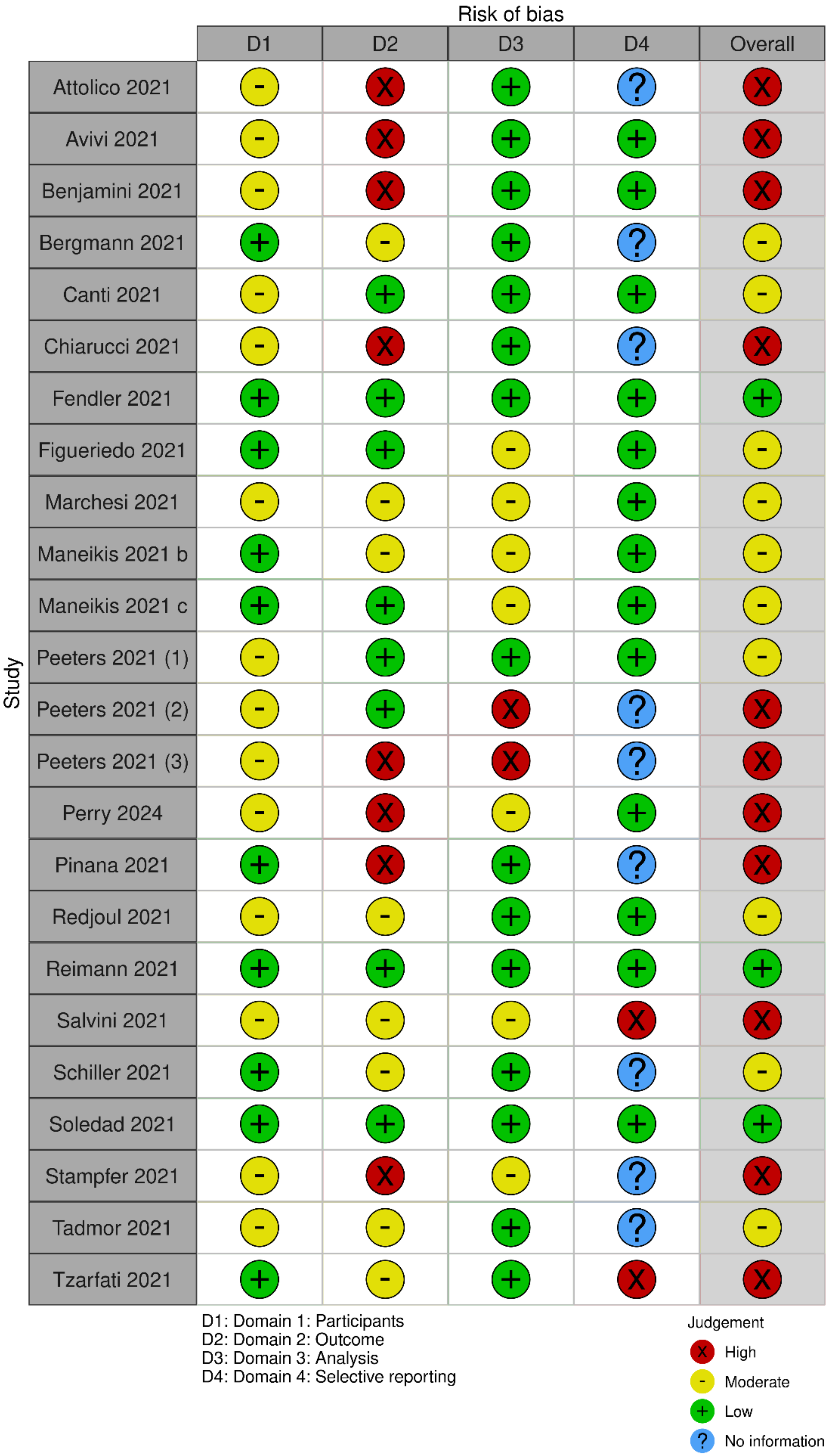
Risk of bias
D1
D2
D3
D4
Overall
Study
Attolico 2021
Avivi 2021
Benjamini 2021
Bergmann 2021
Canti 2021
Chiarucci 2021
Fendler 2021
Figueriedo 2021
Marchesi 2021
Maneikis 2021 b
Maneikis 2021 c
Peeters 2021 (1)
Peeters 2021 (2)
Peeters 2021 (3)
Perry 2024
Pinana 2021
Redjoul 2021
Reimann 2021
Salvini 2021
Schiller 2021
Soledad 2021
Stampfer 2021
Tadmor 2021
Tzarfati 2021
D1: Domain 1: Participants
D2: Domain 2: Outcome
D3: Domain 3: Analysis
D4: Domain 4: Selective reporting
Judgement
High
Moderate
Low
No information
