# Supplementary material for: Effectiveness, immunogenicity, and safety of COVID-19 vaccines for individuals with hematological malignancies: a systematic review
Source: Blood Cancer J. 2022 May 31;12(5):86. doi: 10.1038/s41408-022-00684-8 (PMC9152308; doi:10.1038/s41408-022-00684-8)
Supplement: Supplementary file 5 — supplementary figure 2 [file 41408_2022_684_MOESM5_ESM.pdf]

Risk of bias

Study	D1	D2	D3	D4	Overall
Addeo 2021	-	+	-	+	-
Aleman 2021	-	+	-	+	-
Attolico 2021	-	+	+	?	-
Avivi 2021	-	+	+	+	-
Benjamini 2021 (1)	-	X	+	+	X
Benjamini 2021 (2)	-	X	X	?	X
Bergmann 2021	+	+	-	+	-
Bitoun 2021	-	+	+	+	-
Canti 2021	-	+	+	+	-
Cattaneo 2021	-	X	+	?	X
Chan 2021	-	-	+	+	-
Chiarucci 2021	-	+	+	+	-
Cohen 2021	+	-	+	+	-
Crombie 2021	+	+	+	+	+
Del Poeta 2021	+	+	+	+	+
Ehmsen 2021	X	+	-	+	X
Fendler 2021	+	+	X	+	X
Figueriedo 2021	+	+	-	+	-
Fox 2021	-	-	X	?	X
Gavriatopoulou 2021	+	+	+	+	+
Ghady 2021	-	+	+	?	-
Ghione 2021	+	+	+	+	+
Greenberger 2021	+	+	+	+	+
Gurion 2021	-	+	-	?	-
Jurgens 2021	-	+	+	+	-
Kozak 2021	-	+	+	+	-
Lim 2021	-	-	-	+	-
Lindemann 2021	-	+	-	+	-
Lockmer 2021	-	-	-	+	-
Madhumita 2021	+	-	-	+	-
Mairhofer 2021	-	+	+	+	-
Maneikis 2021	+	+	X	+	X
Marasco 2021 (1)	-	+	X	+	X
Marasco 2021 (2)	-	+	+	+	-
Marchesi 2021	-	+	-	+	-
McKenzie 2021 (1)	+	-	+	+	-
McKenzie 2021 (2)	+	+	+	+	+
Molica 2021	-	+	+	+	-
Mona 2021	+	+	-	+	-
Perry 2021	-	+	+	+	-
Pinana 2021	+	X	+	+	X
Rahav 2021	+	-	+	+	-
Ram 2021	-	+	+	+	-
Ramasamy 2021	+	+	+	+	+
Redjoul 2021	-	+	+	+	-
Reimann 2021	+	+	+	+	+
Salvini 2021	-	+	-	X	X
Schiller 2021	+	+	-	+	-
Shem-Tov 2021	+	+	+	?	-
Shen 2021	-	+	X	?	X
Sherman 2021	-	X	-	+	X
Soledad 2021	+	+	-	+	-
Stampfer 2021	-	+	+	+	-
Tadmor 2021	-	-	+	+	-
Tamari 2021	-	+	X	+	X
Terpos 2021a	+	+	+	+	+
Terpos 2021b	+	+	+	+	+
Tzarfati 2021	+	+	+	?	-
Yeshurun 2021	-	+	X	+	X
Zeng 2021	-	+	+	+	-

Study

D1: Domain 1: Participants
D2: Domain 2: Outcome
D3: Domain 3: Analysis
D4: Domain 4: Selective reporting

Judgement
X High
- Moderate
+ Low
? No information
