# Supplementary material for: Effectiveness, immunogenicity, and safety of COVID-19 vaccines for individuals with hematological malignancies: a systematic review
Source: Blood Cancer J. 2022 May 31;12(5):86. doi: 10.1038/s41408-022-00684-8 (PMC9152308; doi:10.1038/s41408-022-00684-8)
Supplement: Supplementary file 6 — supplementary figure 3 [file 41408_2022_684_MOESM6_ESM.pdf]

Risk of bias

Study	D1	D2	D3	D4	Overall
Avivi 2021	-	-	+	+	-
Benjamini 2021	-	-	+	+	-
Bergmann 2021	+	X	+	?	X
Canti 2021	-	X	+	+	X
Cattaneo 2021	-	X	+	?	X
Eifer 2021	+	-	+	+	-
Figueriedo 2021	+	X	+	+	X
Maneikis 2021 d	+	X	-	?	X
Molica 2021	-	-	+	+	-
Perry 2021	-	X	X	X	X
Pinana 2021	+	X	+	+	X
Rahav 2021 (1)	+	X	+	+	X
Rahav 2021 (2)	+	-	+	+	-
Ram 2021	-	+	+	+	-
Reimann 2021	+	-	+	+	-
Schiller 2021	+	-	+	?	-
Shem-Tov 2021	+	-	X	?	X
Sherman 2021	-	-	-	+	-
Terpos 2021 a	+	X	+	?	X
Terpos 2021 b	+	X	-	?	X
Yeshurun 2021	-	X	-	X	X

D1: Domain 1: Participants
D2: Domain 2: Outcome
D3: Domain 3: Analysis
D4: Domain 4: Selective reporting

Judgement
X High
- Moderate
+ Low
? No information
