# Supplementary figures and images for: Effectiveness, immunogenicity, and safety of COVID-19 vaccines for individuals with hematological malignancies: a systematic review
Source: Blood Cancer J. 2022 May 31;12(5):86. doi: 10.1038/s41408-022-00684-8 (PMC9152308; doi:10.1038/s41408-022-00684-8)

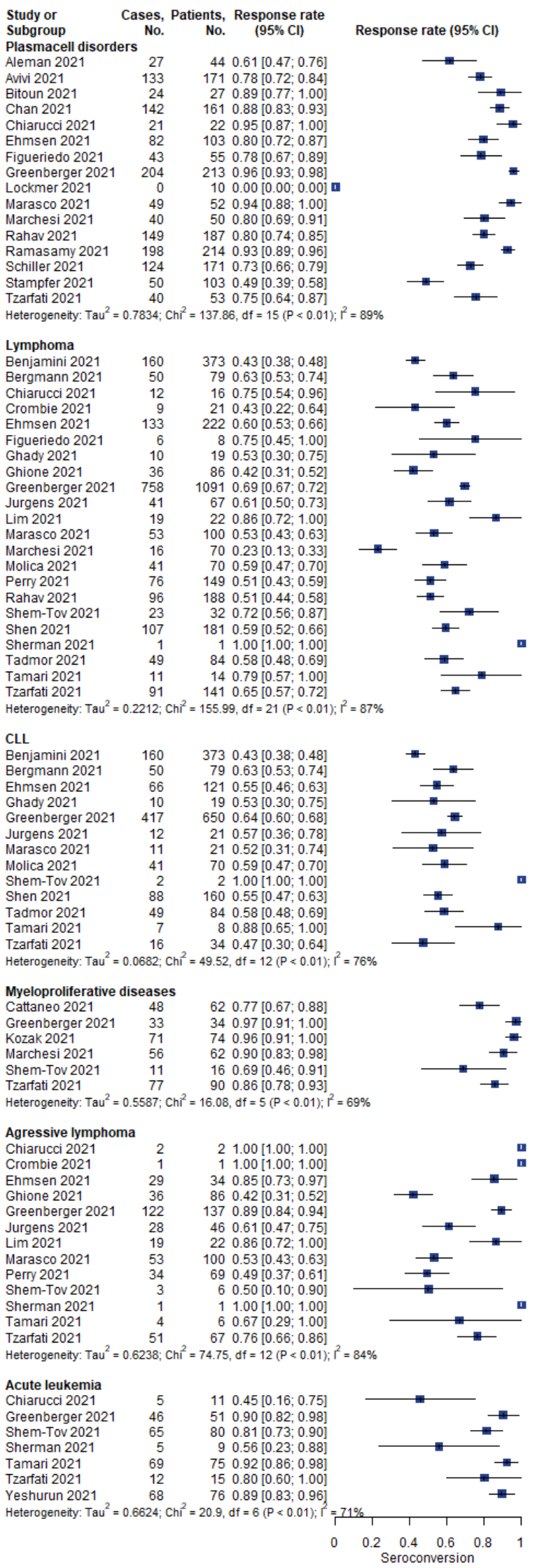

Supplement: Supplementary file 7 — supplementary figure 4 [file 41408_2022_684_MOESM7_ESM.pdf]

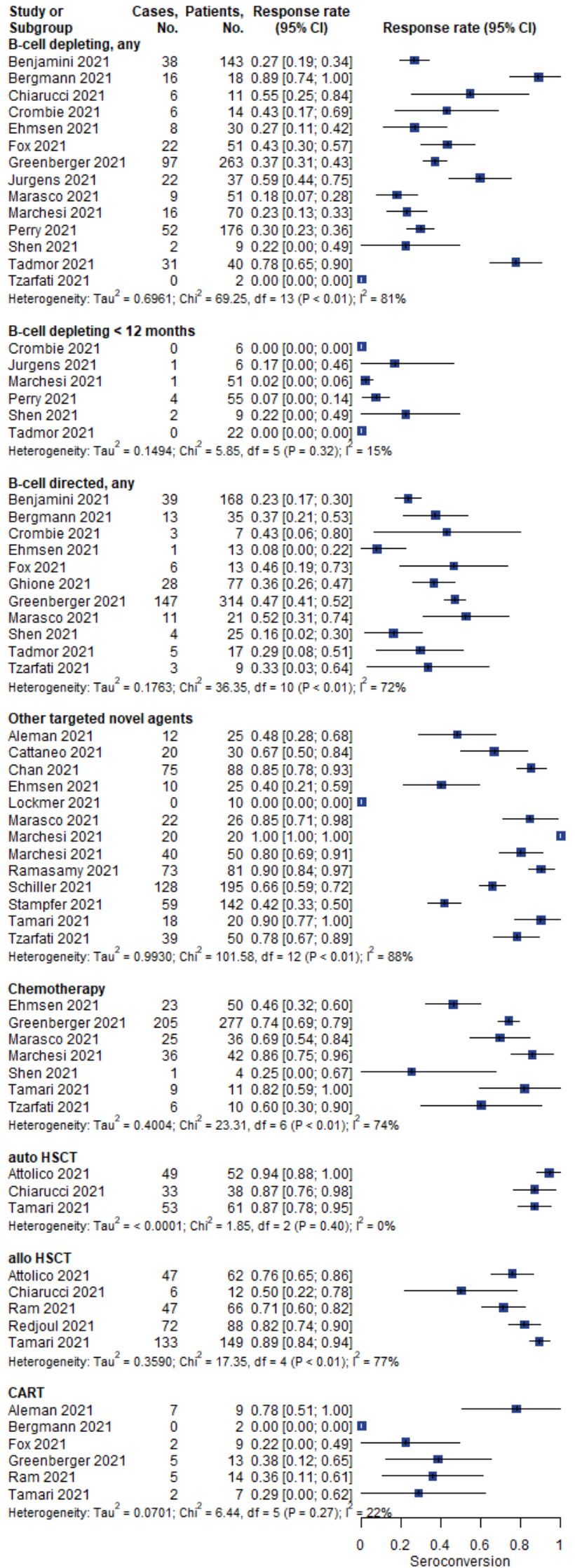

Supplement: Supplementary file 8 — supplementary figure 5 [file 41408_2022_684_MOESM8_ESM.pdf]

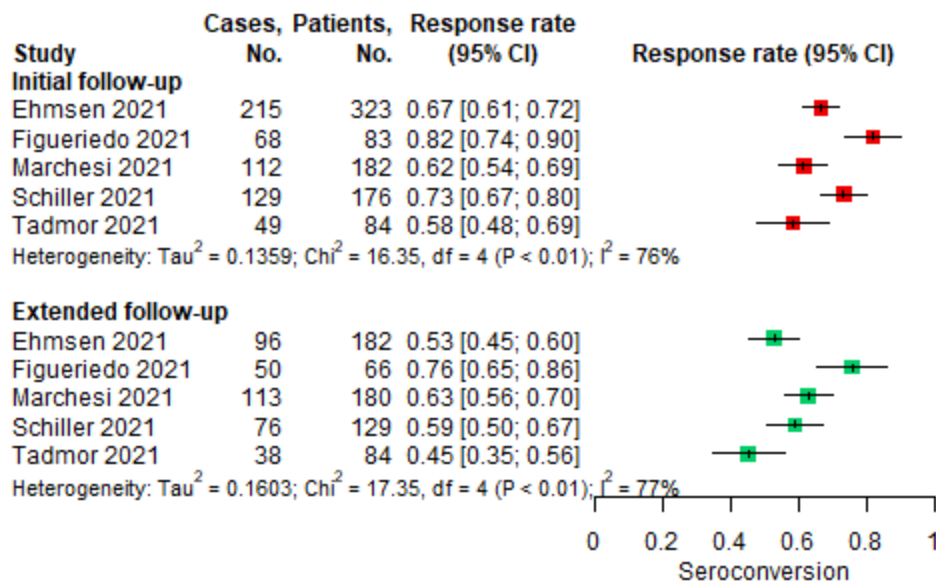

Supplement: Supplementary file 9 — supplementary figure 6 [file 41408_2022_684_MOESM9_ESM.pdf]

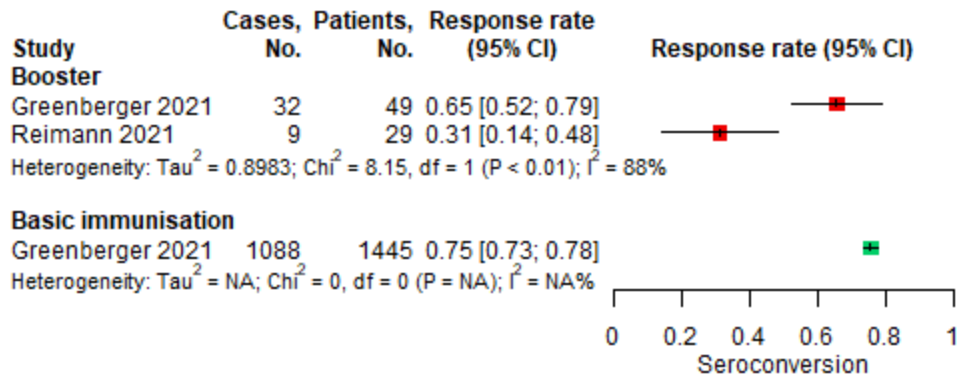

Supplement: Supplementary file 10 — supplementary figure 7 [file 41408_2022_684_MOESM10_ESM.pdf]

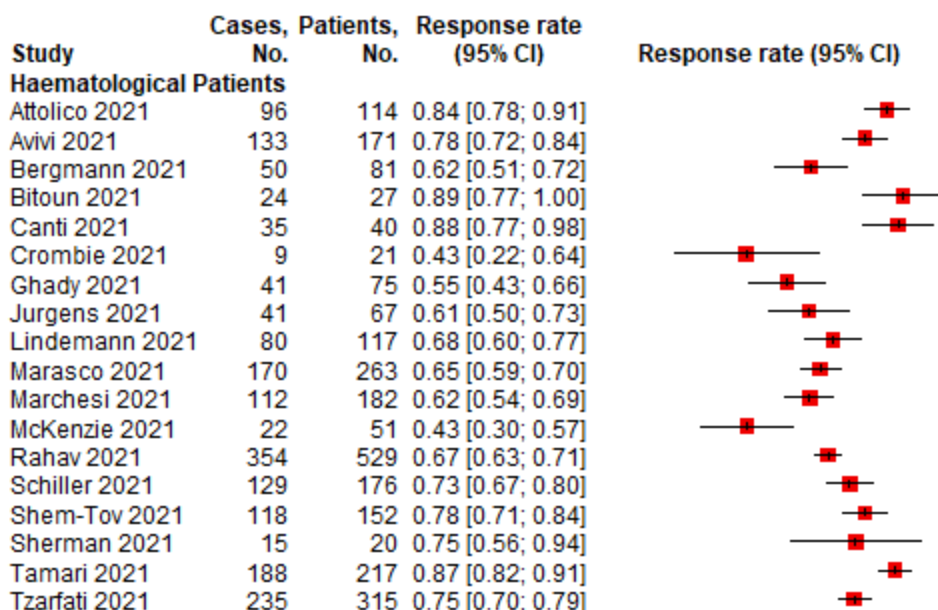

Heterogeneity:  $\tau^2 = 0.3182$ ;  $\chi^2 = 107$ ,  $df = 17$  ( $P < 0.01$ );  $I^2 = 84\%$

#### Healthy control

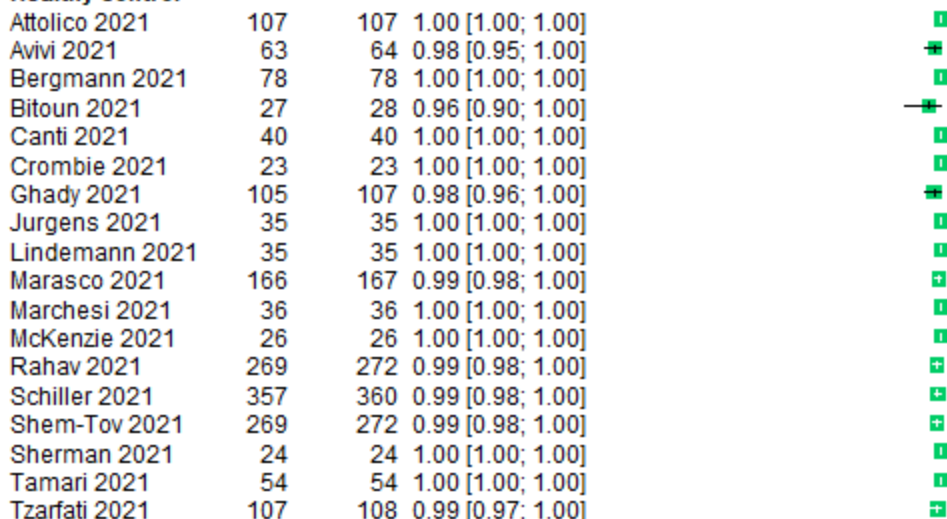

Heterogeneity:  $\tau^2 = 0$ ;  $\chi^2 = 3.78$ ,  $df = 17$  ( $P = 1.00$ );  $I^2 = 0\%$

Supplement: Supplementary file 12 — supplementary figure 9 [file 41408_2022_684_MOESM12_ESM.pdf]
