# Supplementary material for: Effectiveness, immunogenicity, and safety of COVID-19 vaccines for individuals with hematological malignancies: a systematic review
Source: Blood Cancer J. 2022 May 31;12(5):86. doi: 10.1038/s41408-022-00684-8 (PMC9152308; doi:10.1038/s41408-022-00684-8)

Study	Cases, No.	Patients, No.	Response rate (95% CI)
**BNT162b2**			
Cattaneo 2021	19	22	0.86 [0.72; 1.00]
Chan 2021	103	118	0.87 [0.81; 0.93]
Mairhofer 2021	3	16	0.19 [0.00; 0.38]
Stampfer 2021	13	48	0.27 [0.15; 0.40]
Tamari 2021	129	152	0.85 [0.79; 0.91]
Heterogeneity: $Tau^2 = 2.5709$; $Chi^2 = 75.45$, df = 4 ($P < 0.01$); $I^2 = 95\%$			
**mRNA-1273**			
Cattaneo 2021	29	40	0.72 [0.59; 0.86]
Mairhofer 2021	3	14	0.21 [0.00; 0.43]
Stampfer 2021	30	48	0.62 [0.49; 0.76]
Tamari 2021	59	65	0.91 [0.84; 0.98]
Heterogeneity: $Tau^2 = 1.7683$; $Chi^2 = 23.52$, df = 3 ($P < 0.01$); $I^2 = 87\%$			
**ChAdox-nCoV-19**			
Chan 2021	41	45	0.91 [0.83; 0.99]
Heterogeneity: Tau^2 = NA; $Chi^2 = 0$, df = 0 (P = NA); I^2 = NA%			

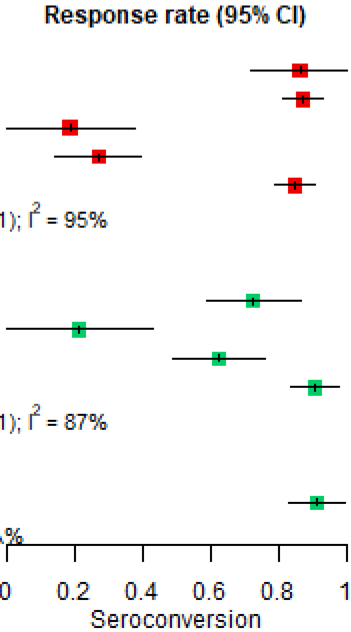

Supplement: Supplementary file 11 — supplementary figure 8 [file 41408_2022_684_MOESM11_ESM.pdf]
